# Supplementary material for: Revealing brain cell-stratified causality through dissecting causal variants according to their cell-type-specific effects on gene expression
Source: Nat Commun. 2024 Jun 7;15:4890. doi: 10.1038/s41467-024-49263-4 (PMC11161590; doi:10.1038/s41467-024-49263-4)
Supplement: Supplementary file 1 — Supplementary Information [file 41467_2024_49263_MOESM1_ESM.pdf]

Supplementary Figures

Revealing brain cell-stratified causality through dissecting causal variants according to their cell-type-specific effects on gene expression

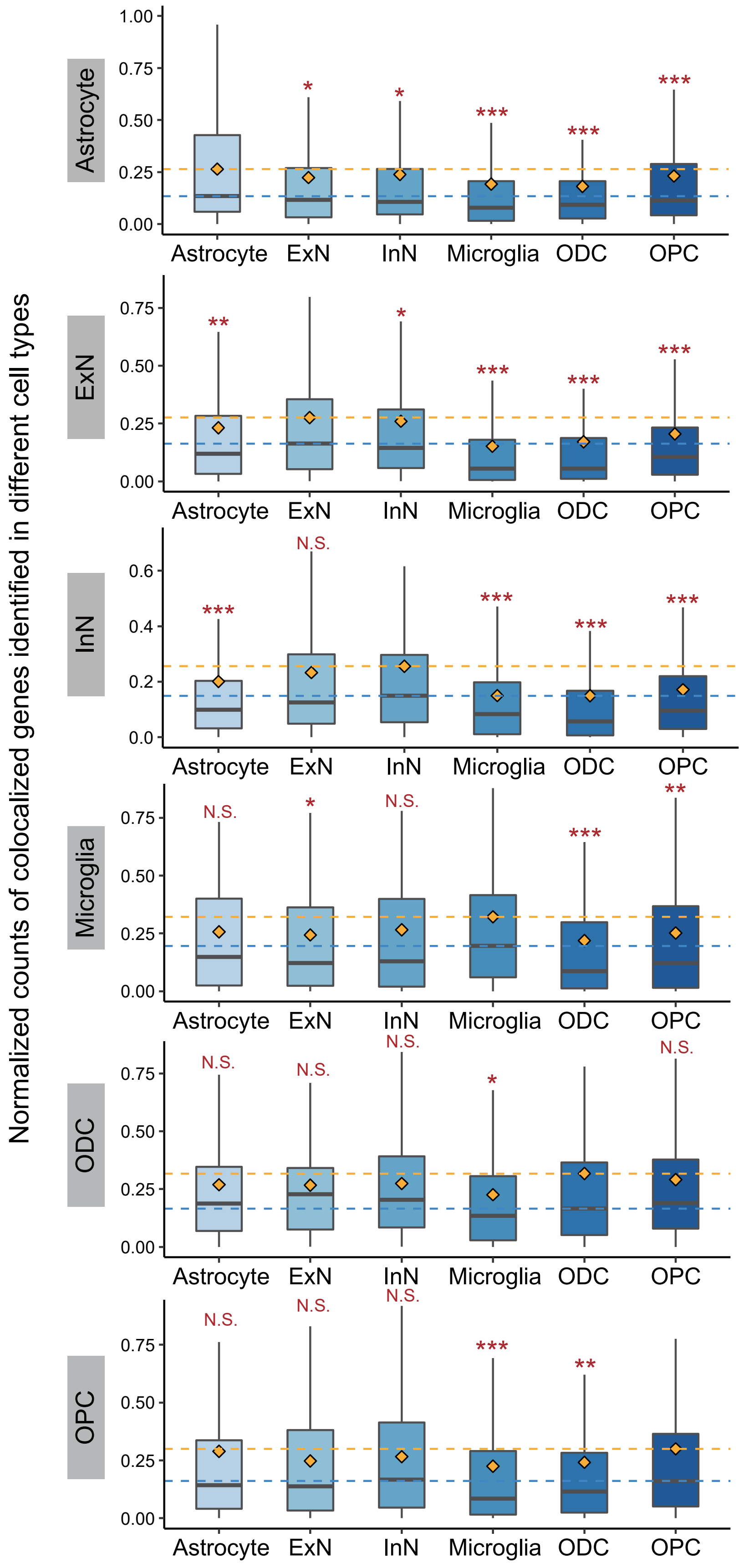

Supplementary Fig. 1. Comparison of normalized expression of colocalized genes across different cell types. The “reference” cell types where genes colocalized are shown on the left. Each box plot shows the distribution of averaged expression of all colocalized genes in the “test” cell type. The box plots represent 25th, 50th, and 75th percentiles, and whiskers extend to 1.5 times the interquartile range. The yellow diamond indicates average expression in each test cell. The yellow and blue dashed lines show the mean and median expression levels in reference cell, respectively. The numbers of tested genes (*N*) are 104, 144, 120, 76, 126, 107, which were identified in reference cell astrocyte, ExN, InN, microglia, ODC and OPC, respectively. *P* values were calculated by two-sided paired Wilcoxon tests (*** $P < 0.001$,** $P < 0.01$, * $P < 0.05$, “N.S.” stands for not significant). The exact *P* values are listed in Supplementary Data 3.

(a) Sleep disorders

Astrocyte

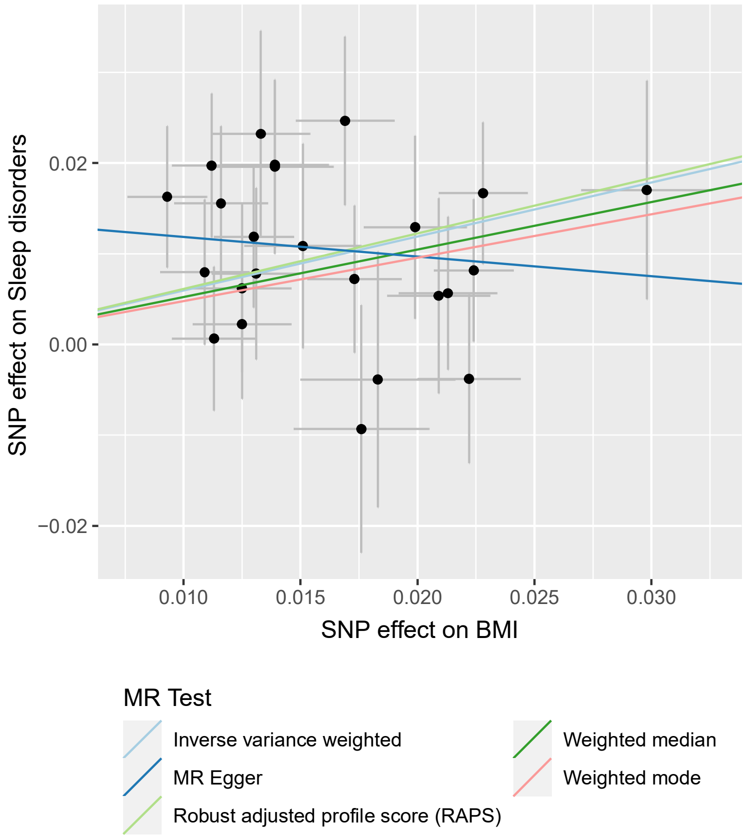

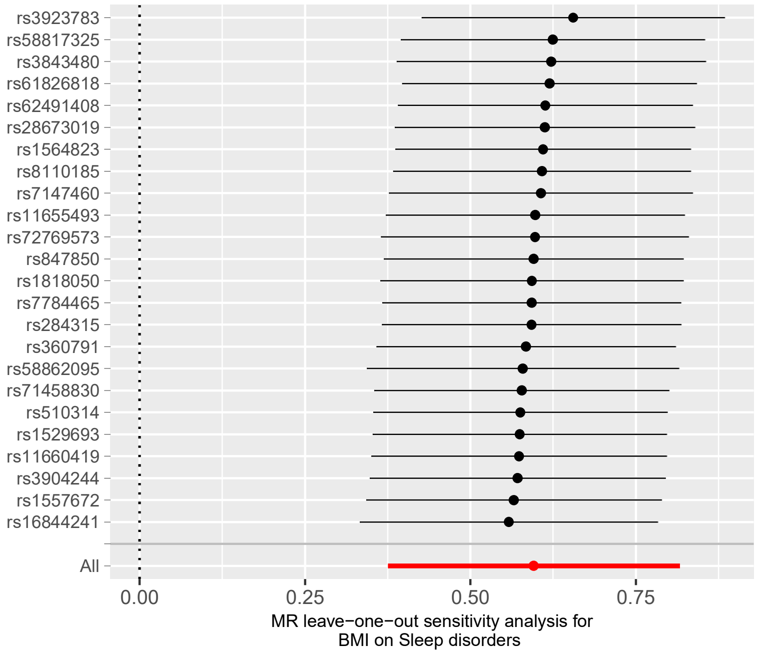

EC

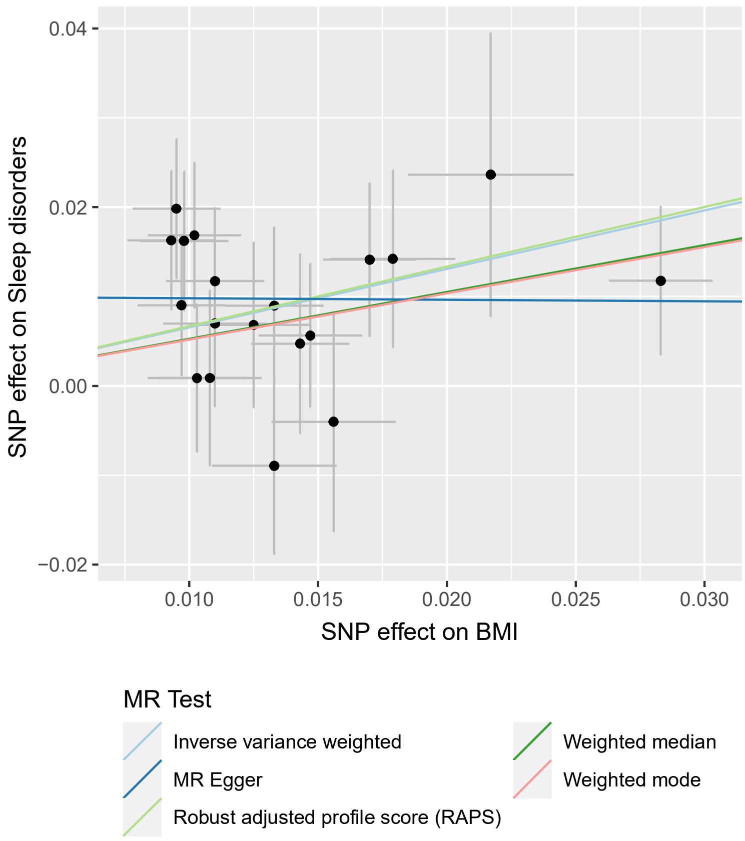

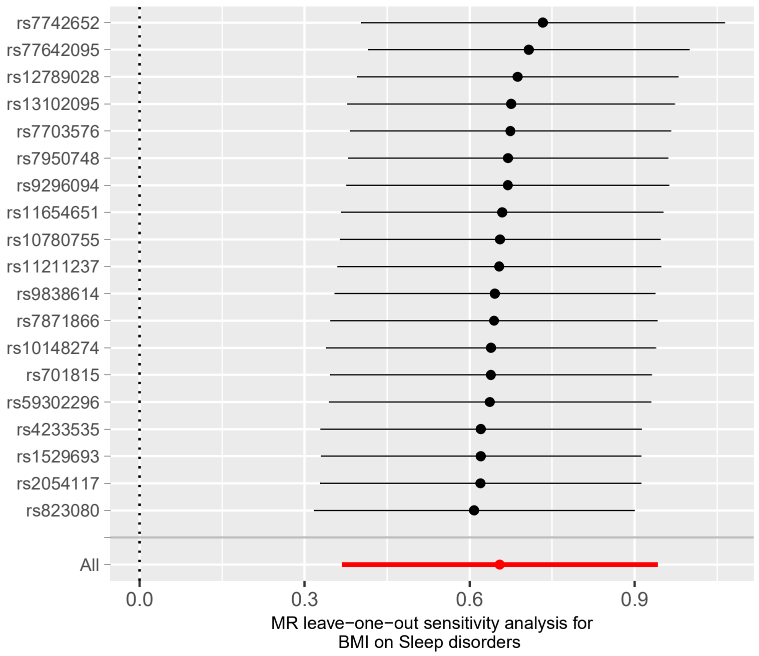

ExN

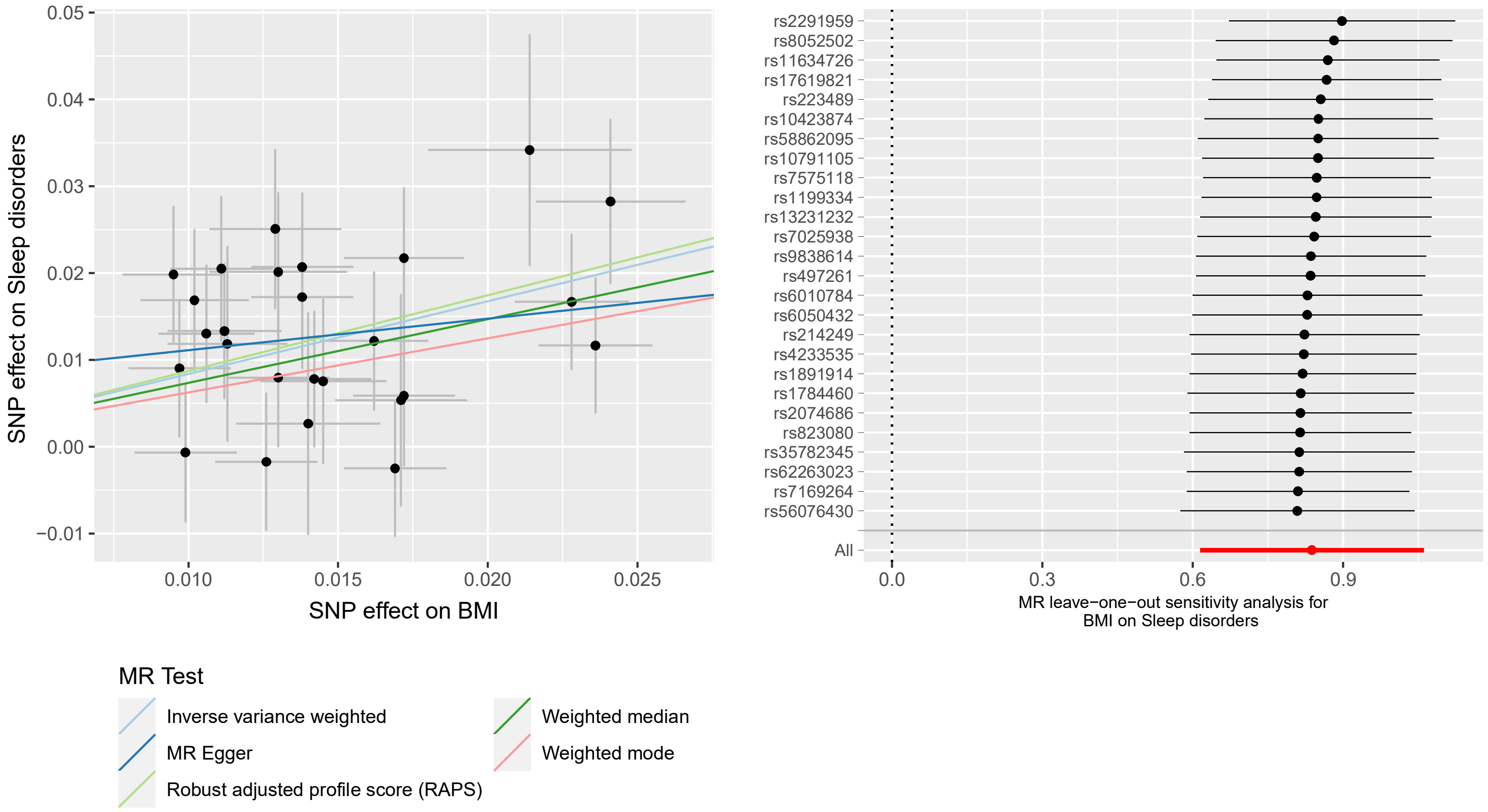

Microglia

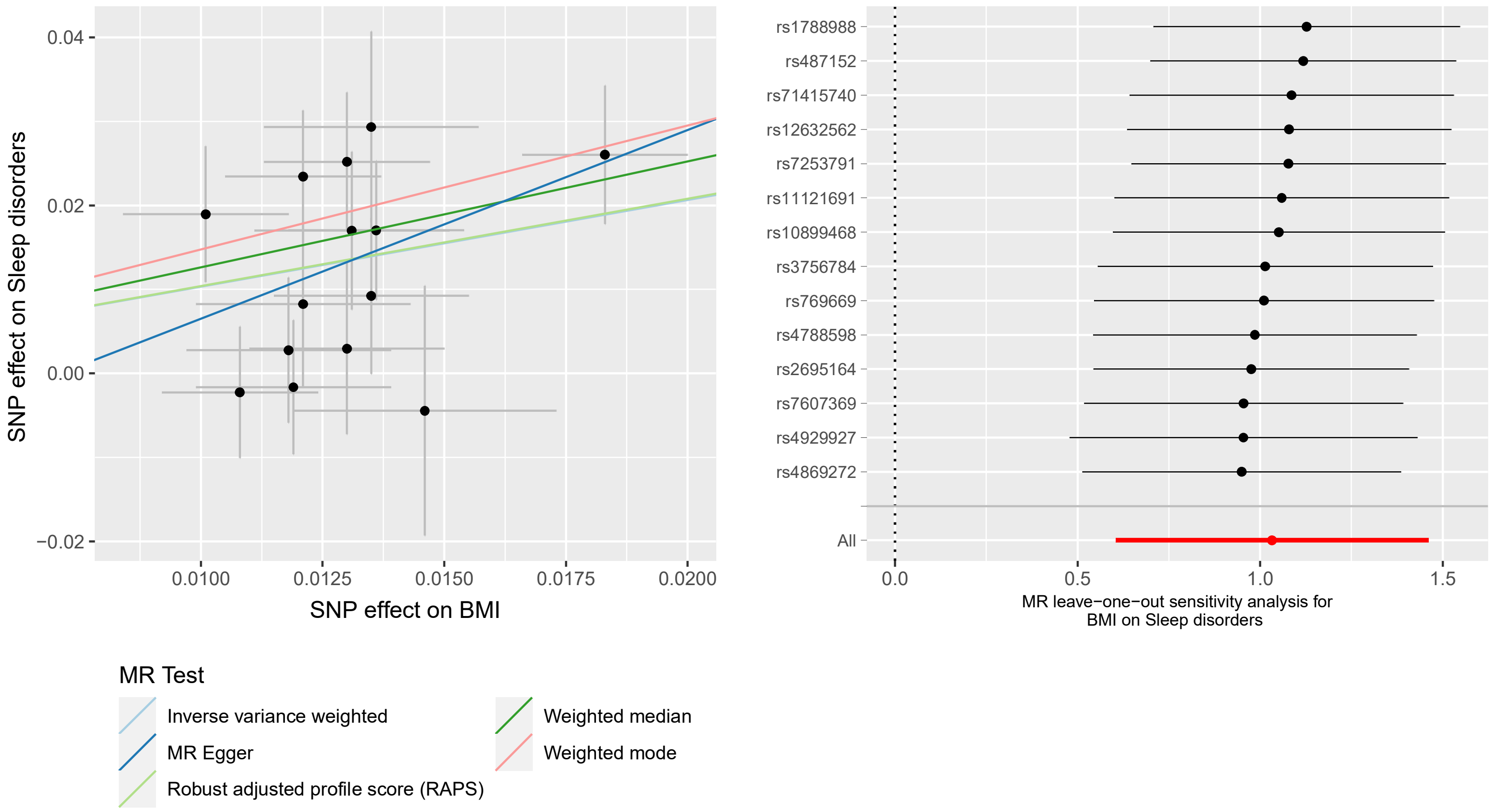

ODC

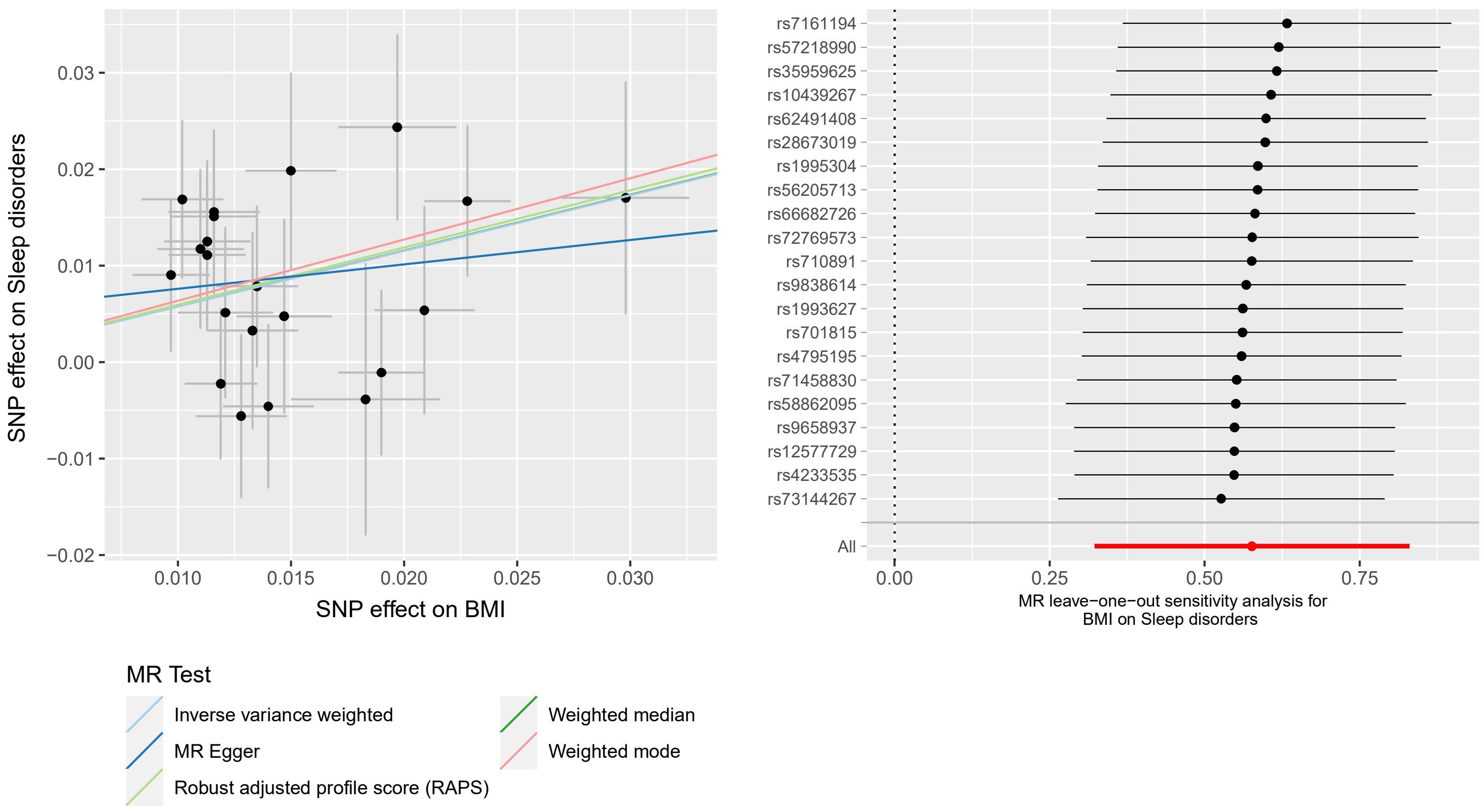
SNP effect on Sleep disorders
SNP effect on BMI
0.03
0.02
0.01
0.00
−0.01
−0.02
0.010
0.015
0.020
0.025
0.030
rs7161194
rs57218990
rs35959625
rs10439267
rs62491408
rs28673019
rs1995304
rs56205713
rs66682726
rs72769573
rs710891
rs9838614
rs1993627
rs701815
rs4795195
rs71458830
rs58862095
rs9658937
rs12577729
rs4233535
rs73144267
All
0.00
0.25
0.50
0.75
MR leave−one−out sensitivity analysis for
BMI on Sleep disorders
MR Test
Inverse variance weighted
MR Egger
Robust adjusted profile score (RAPS)
Weighted median
Weighted mode

OPC

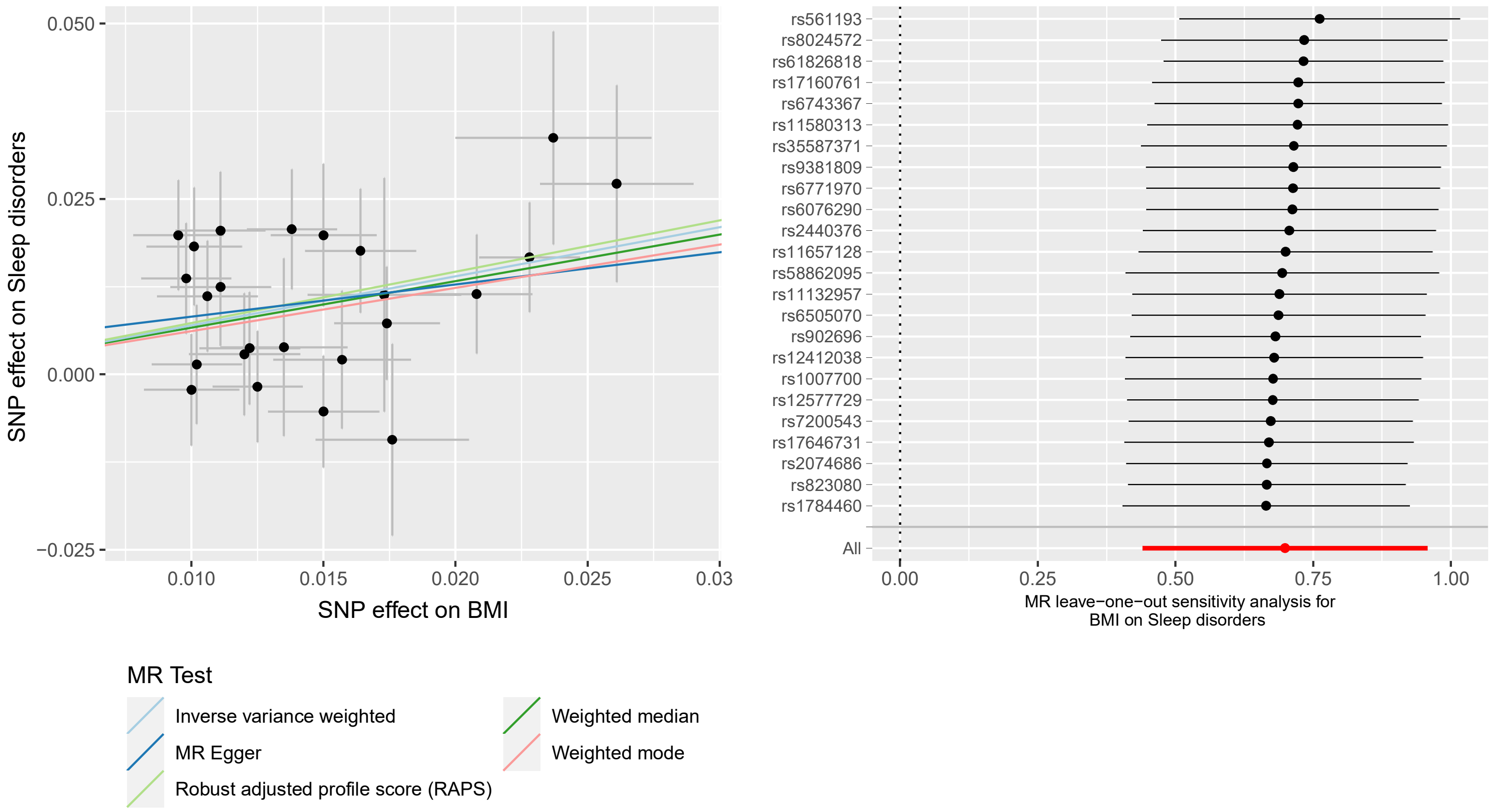
SNP effect on Sleep disorders
SNP effect on BMI
0.050
0.025
0.000
−0.025
0.010
0.015
0.020
0.025
0.03
rs561193
rs8024572
rs61826818
rs17160761
rs6743367
rs11580313
rs35587371
rs9381809
rs6771970
rs6076290
rs2440376
rs11657128
rs58862095
rs11132957
rs6505070
rs902696
rs12412038
rs1007700
rs12577729
rs7200543
rs17646731
rs2074686
rs823080
rs1784460
All
0.00
0.25
0.50
0.75
1.00
MR leave−one−out sensitivity analysis for
BMI on Sleep disorders
MR Test
Inverse variance weighted
MR Egger
Robust adjusted profile score (RAPS)
Weighted median
Weighted mode

(b) ADHD

Astrocyte

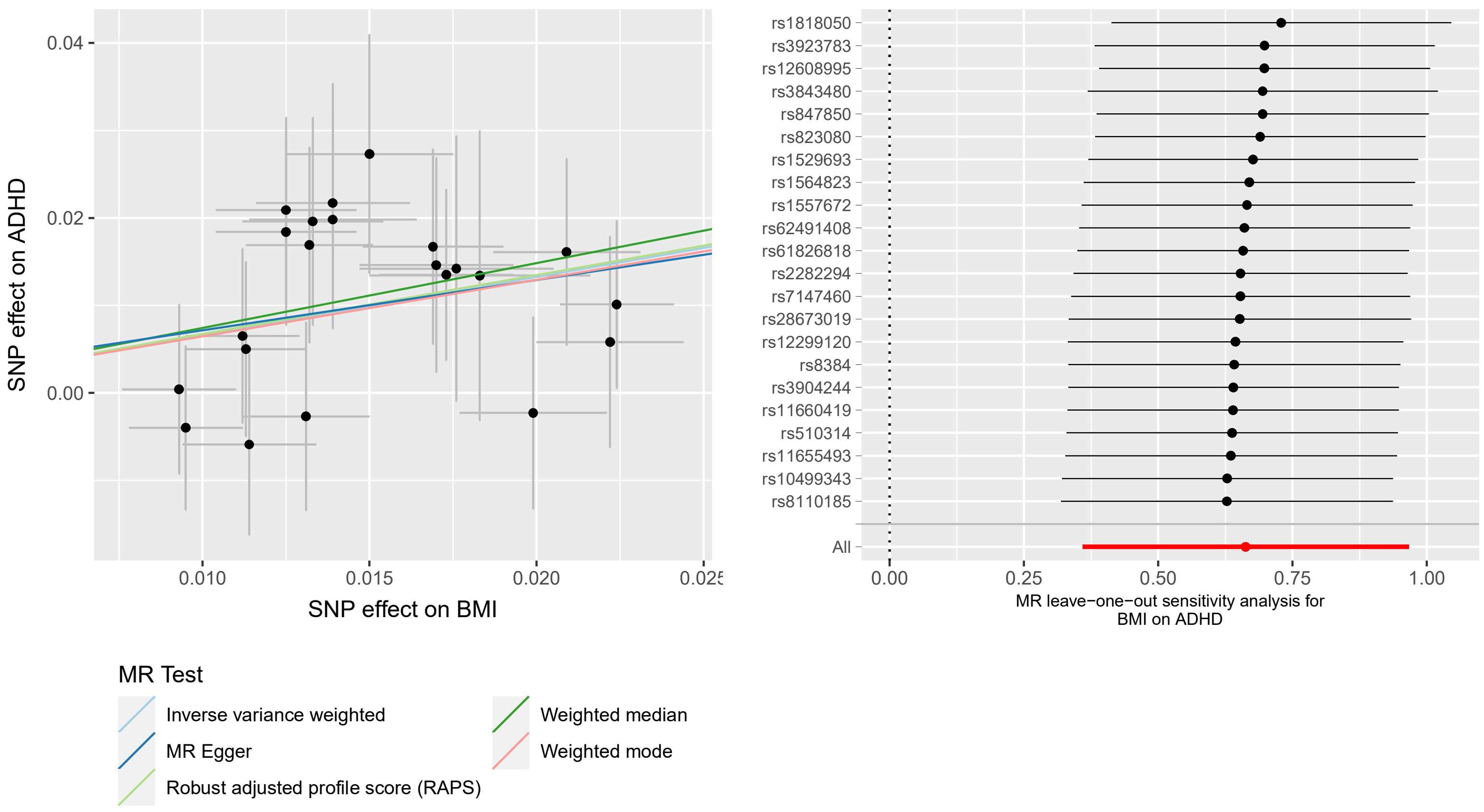

EC

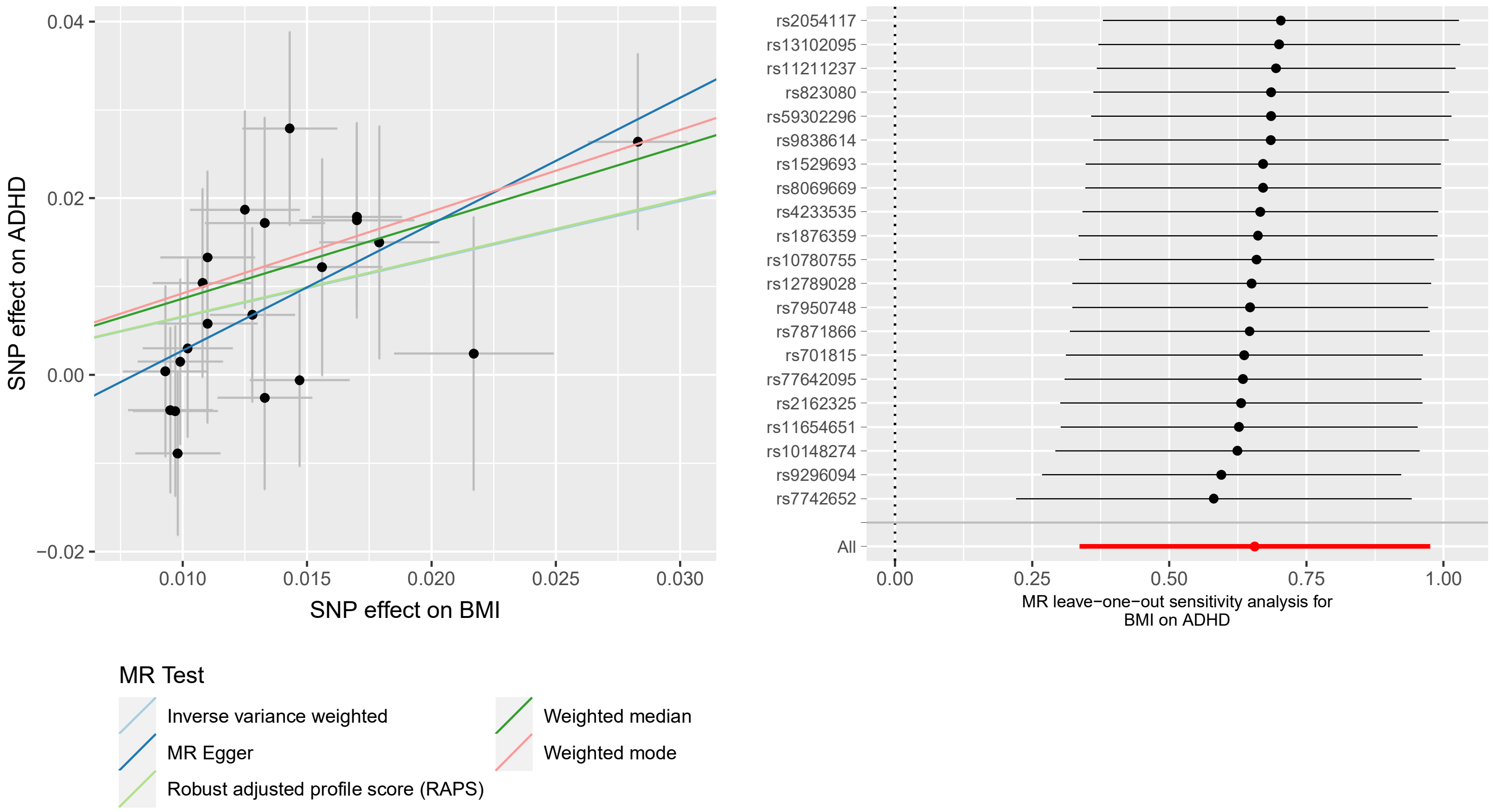

(c) Gout

ExN

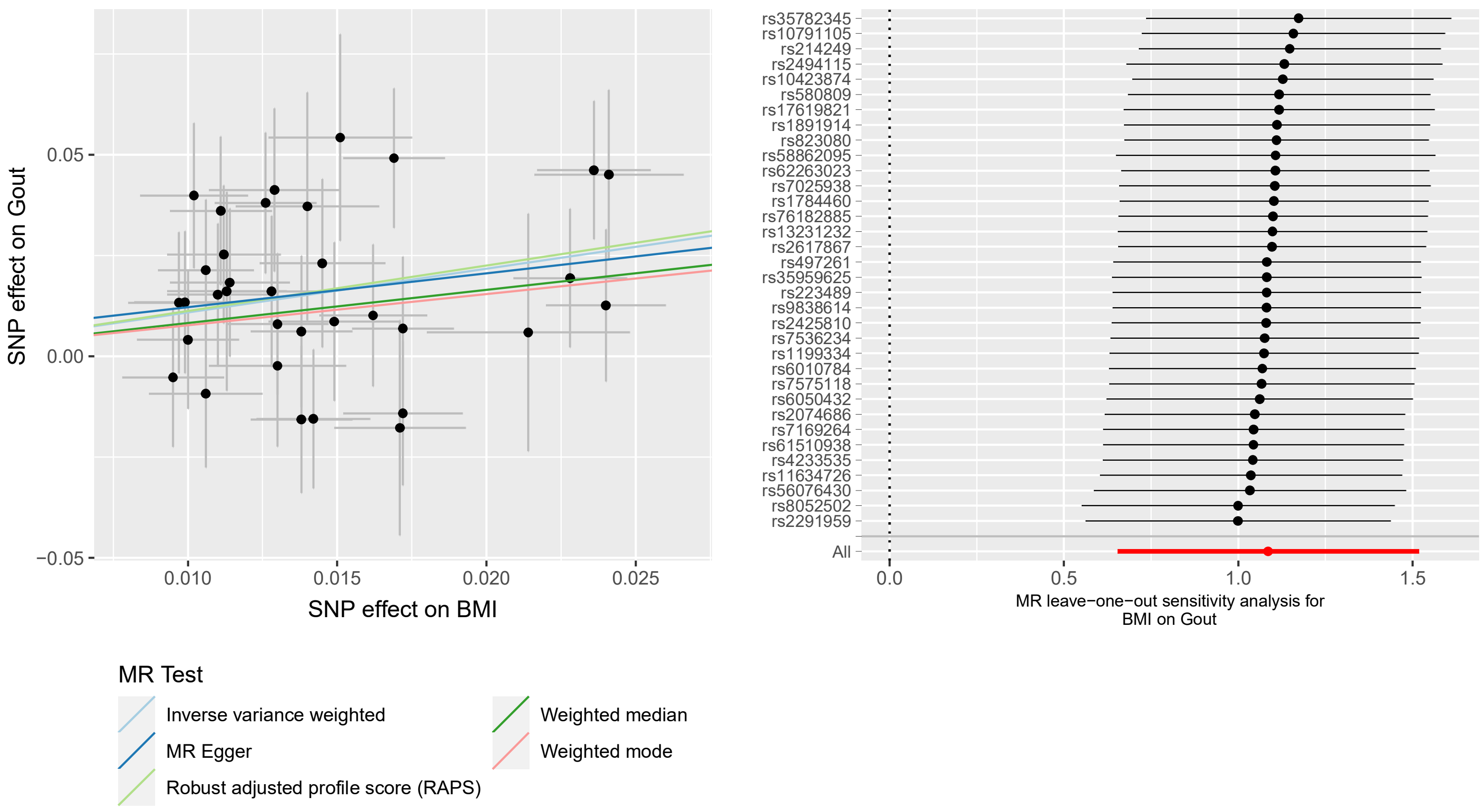

Microglia

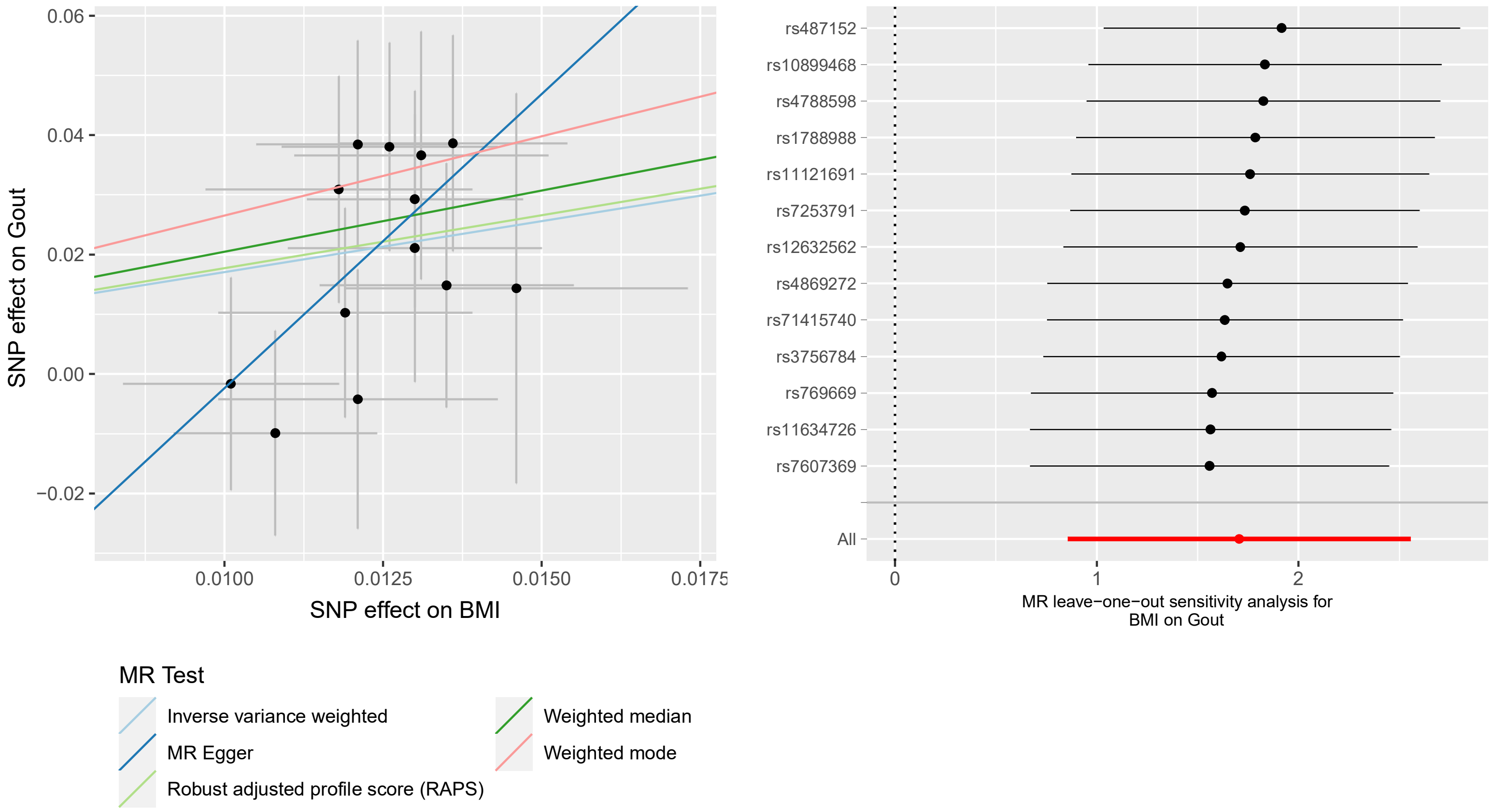

ODC

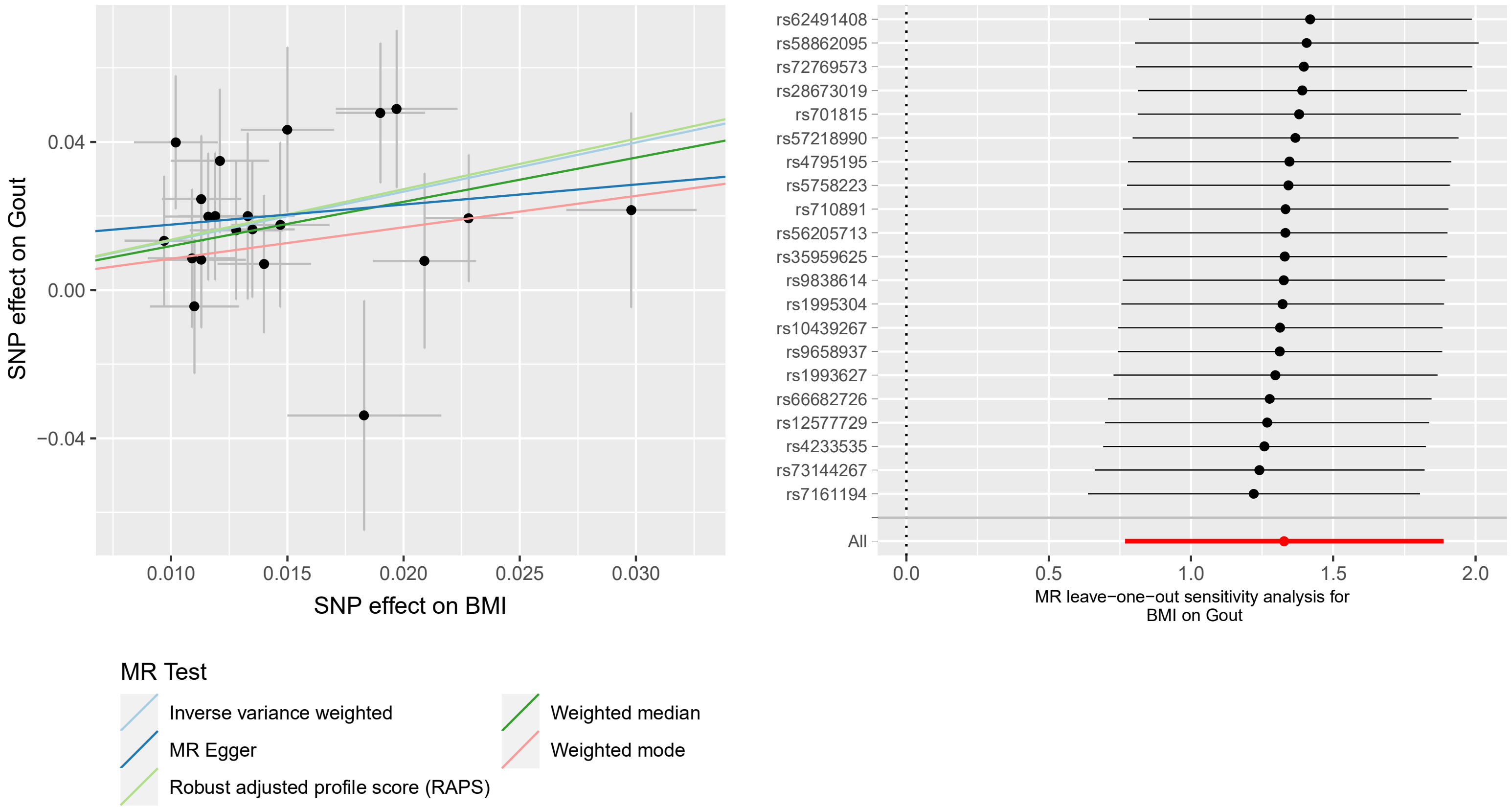
SNP effect on Gout
0.04
0.00
−0.04
0.010
0.015
0.020
0.025
0.030
SNP effect on BMI
rs62491408
rs58862095
rs72769573
rs28673019
rs701815
rs57218990
rs4795195
rs5758223
rs710891
rs56205713
rs35959625
rs9838614
rs1995304
rs10439267
rs9658937
rs1993627
rs66682726
rs12577729
rs4233535
rs73144267
rs7161194
All
0.0
0.5
1.0
1.5
2.0
MR leave−one−out sensitivity analysis for
BMI on Gout
MR Test
Inverse variance weighted
MR Egger
Robust adjusted profile score (RAPS)
Weighted median
Weighted mode

(d) Osteoporosis

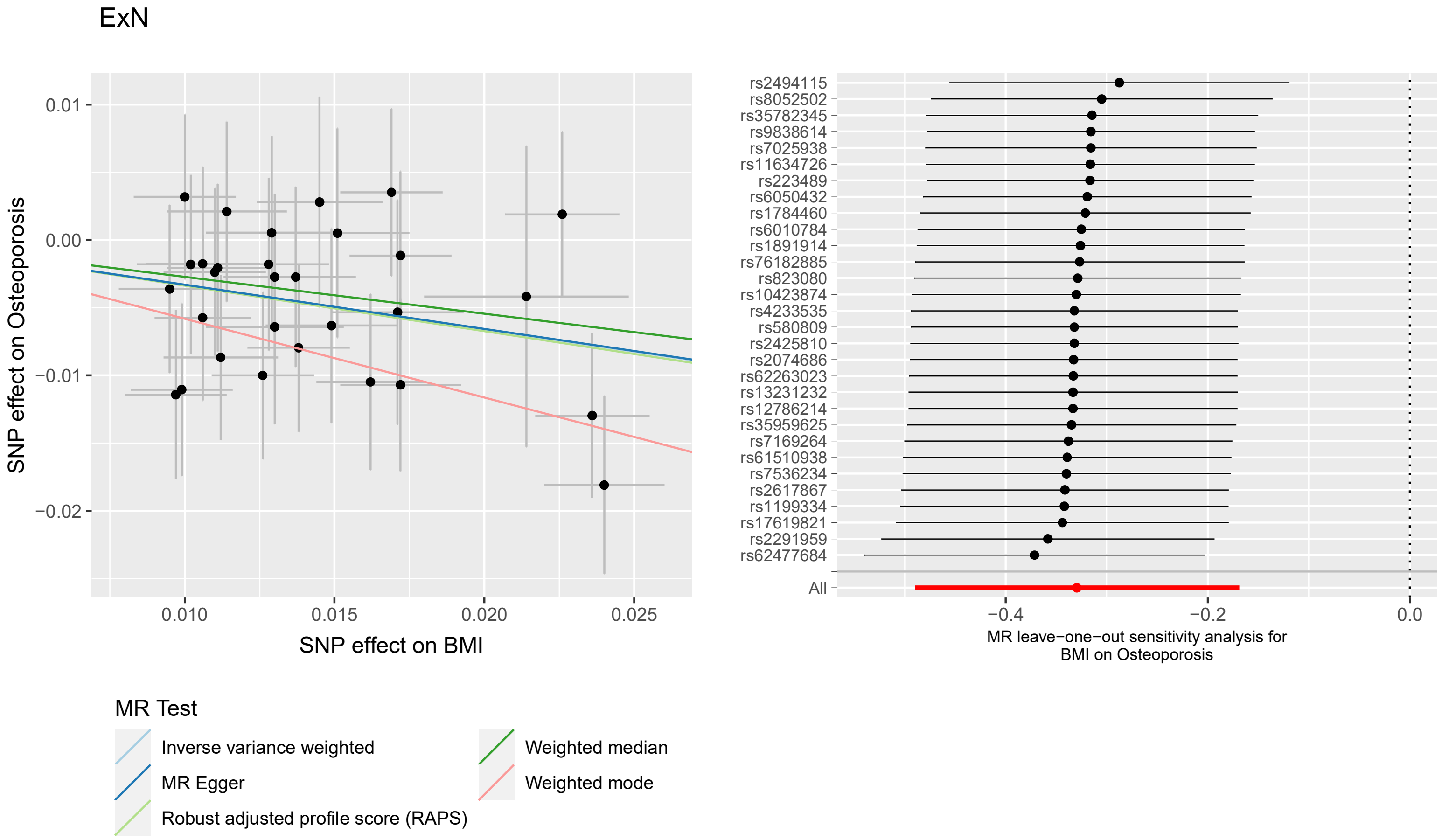

(e) Coronary artery disease

Astrocyte

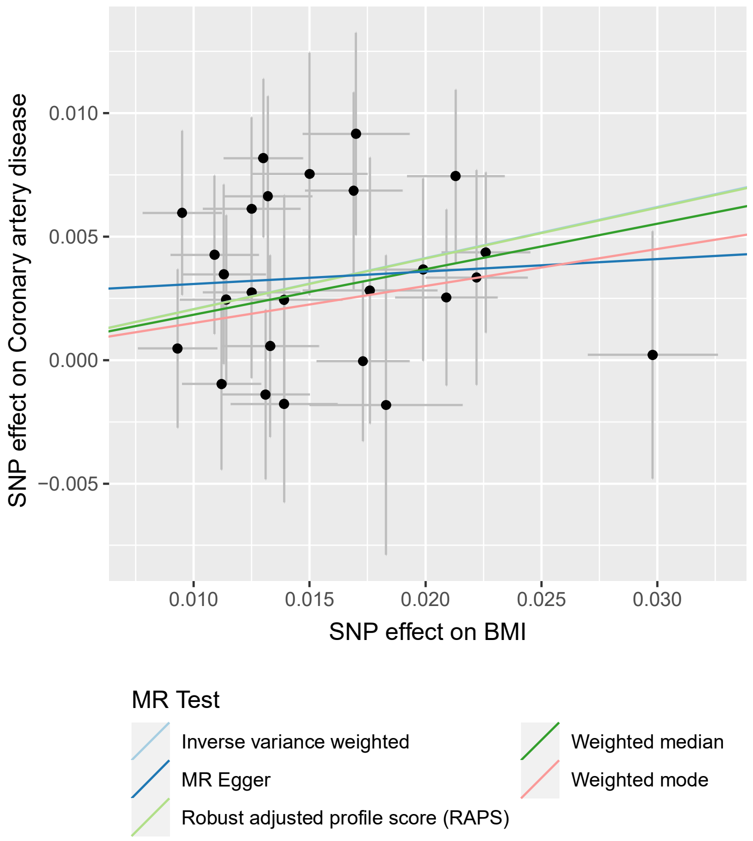

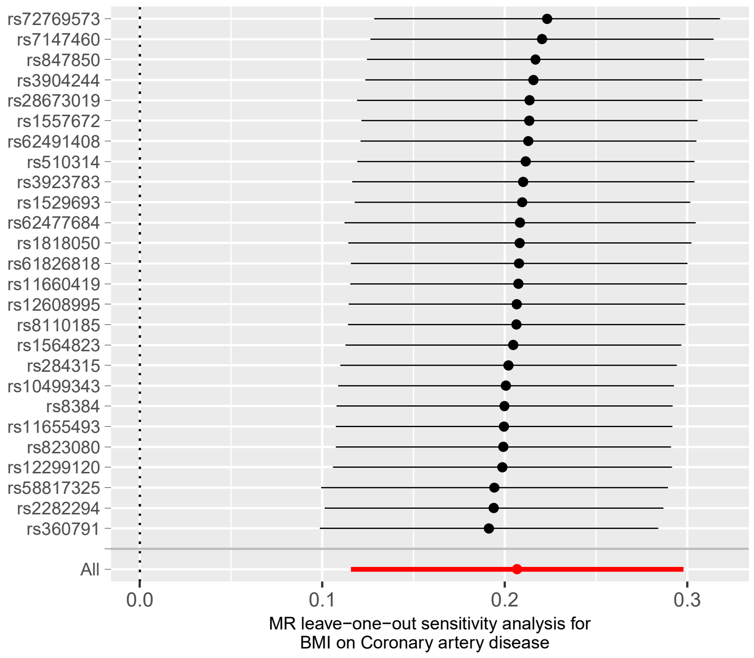

ExN

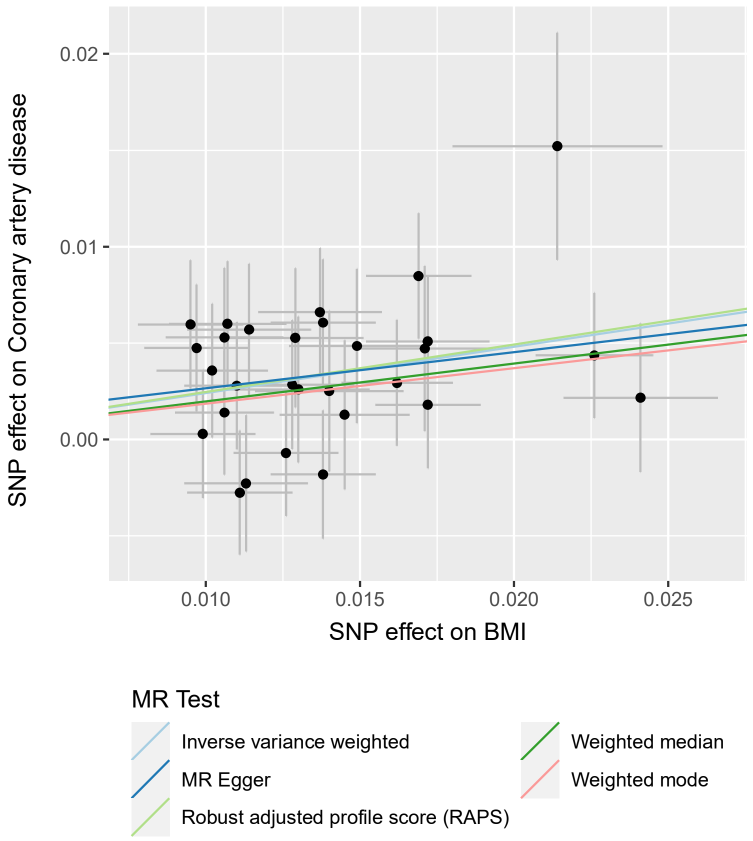

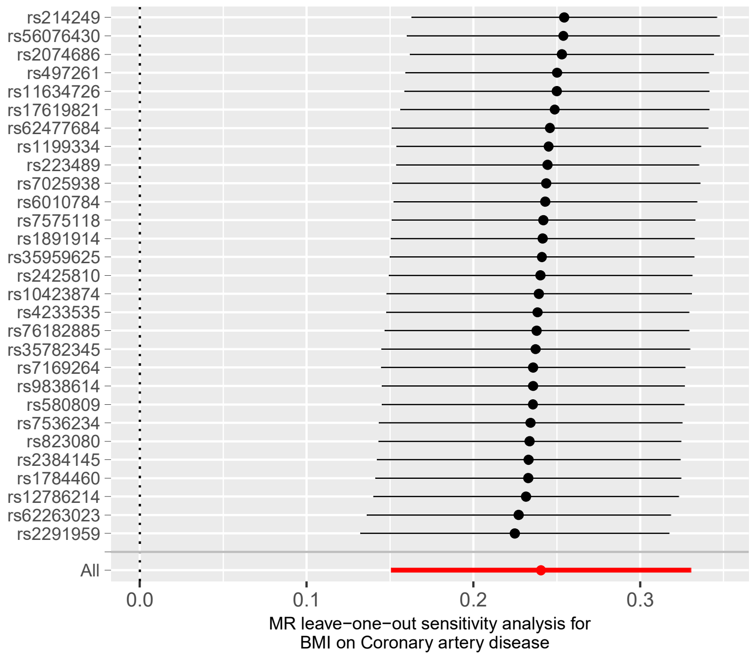

(f) Myocardial infarction

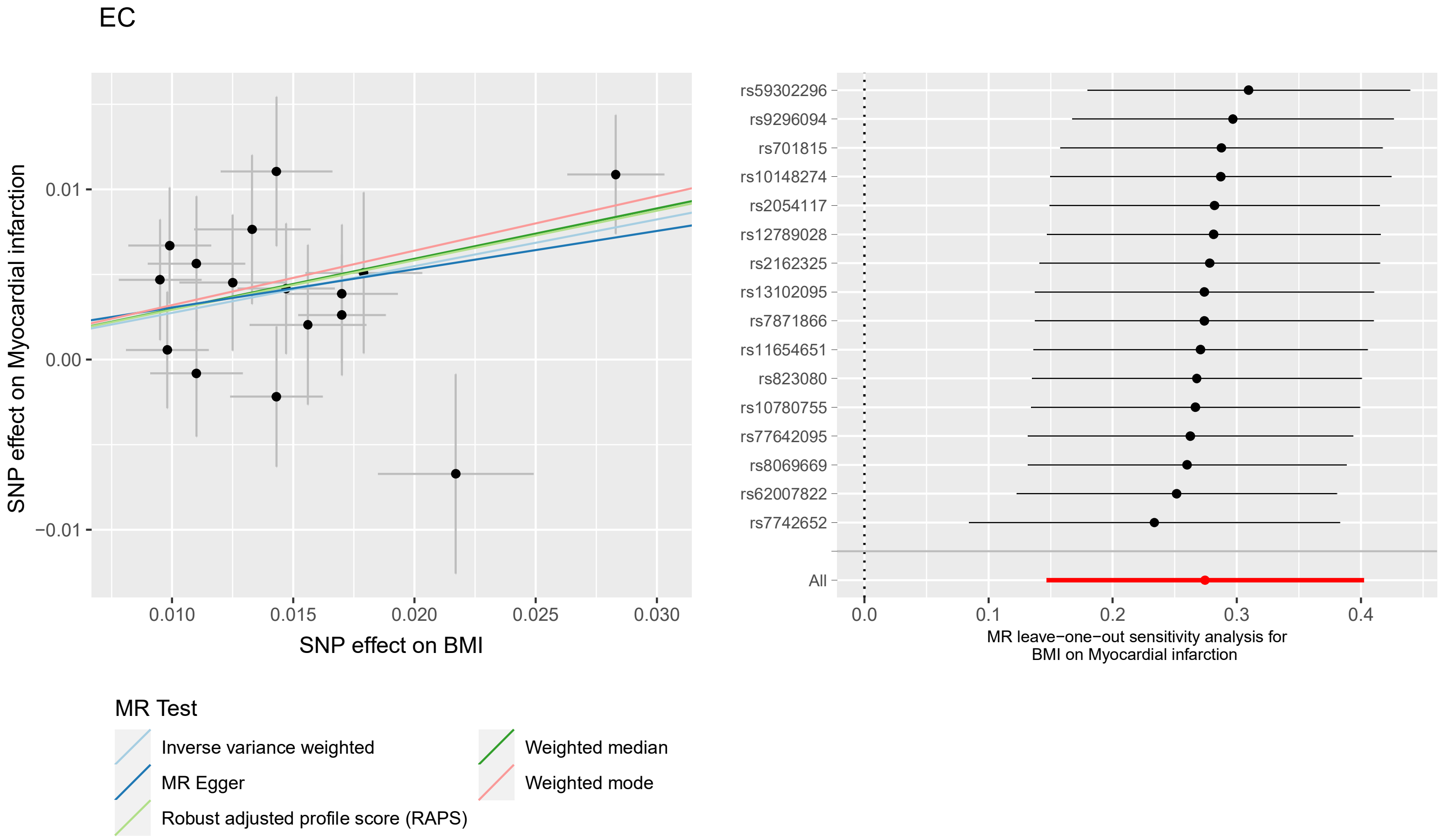

(g) Type II diabetes

Astrocyte

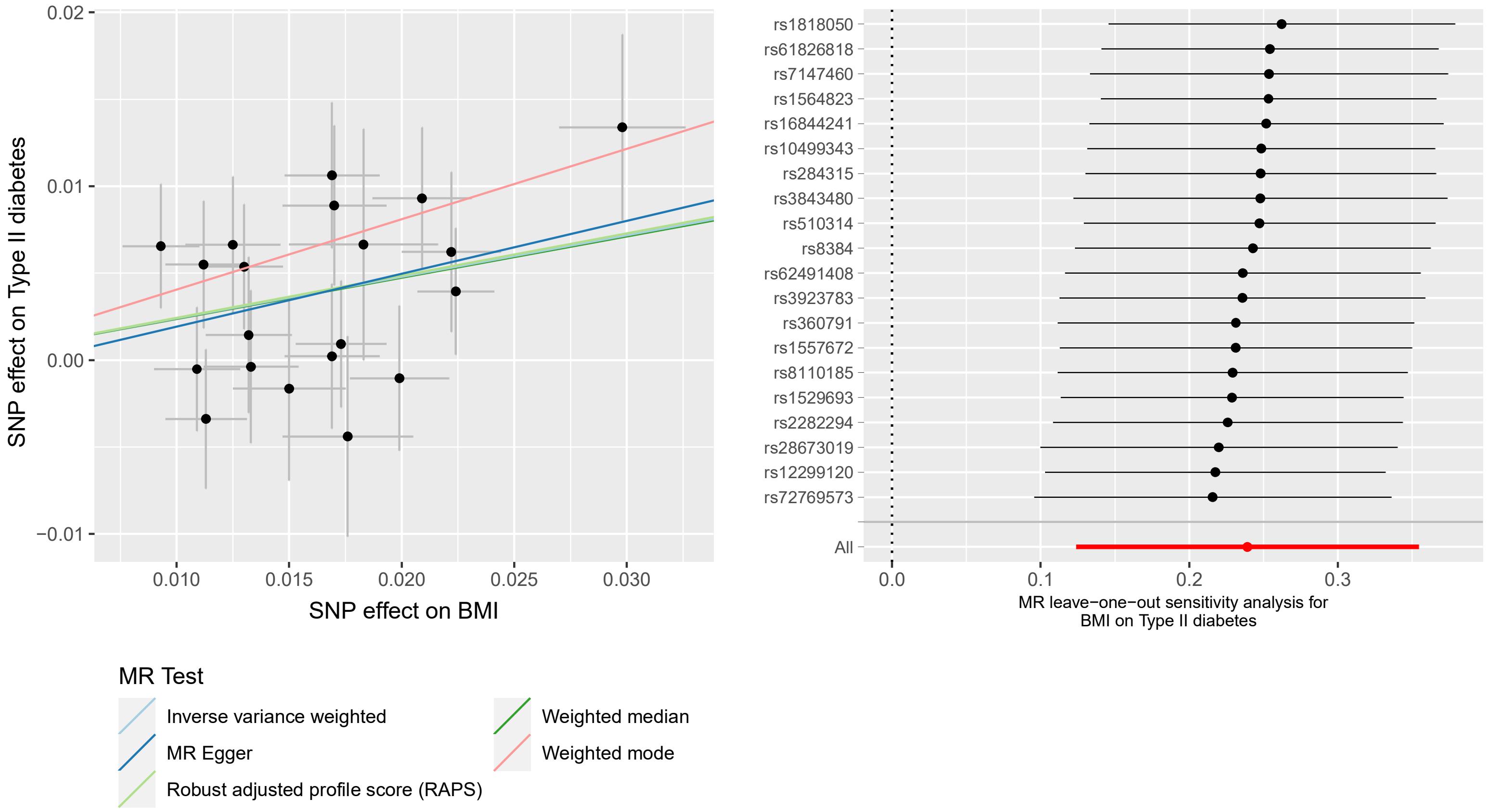

EC

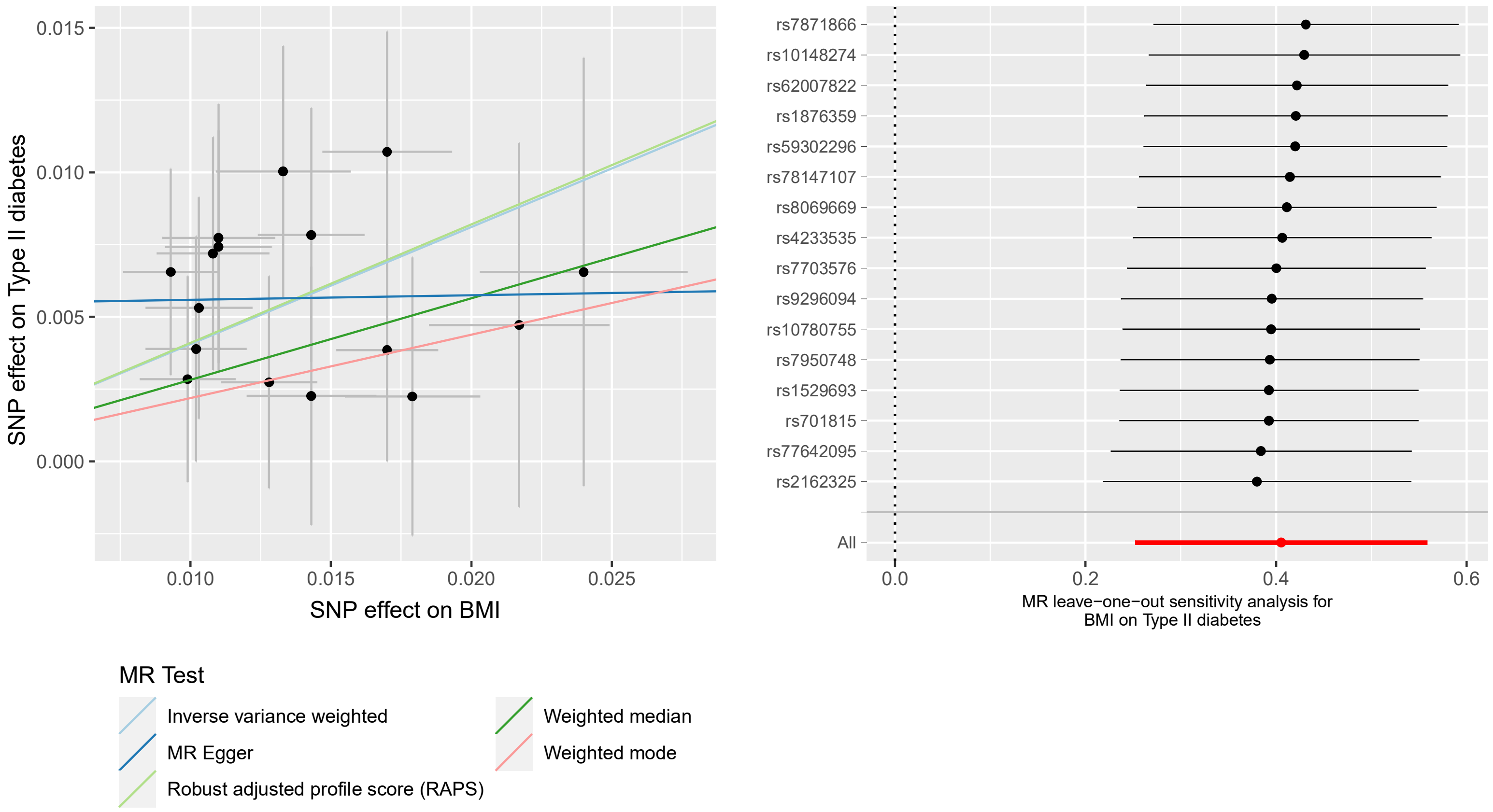

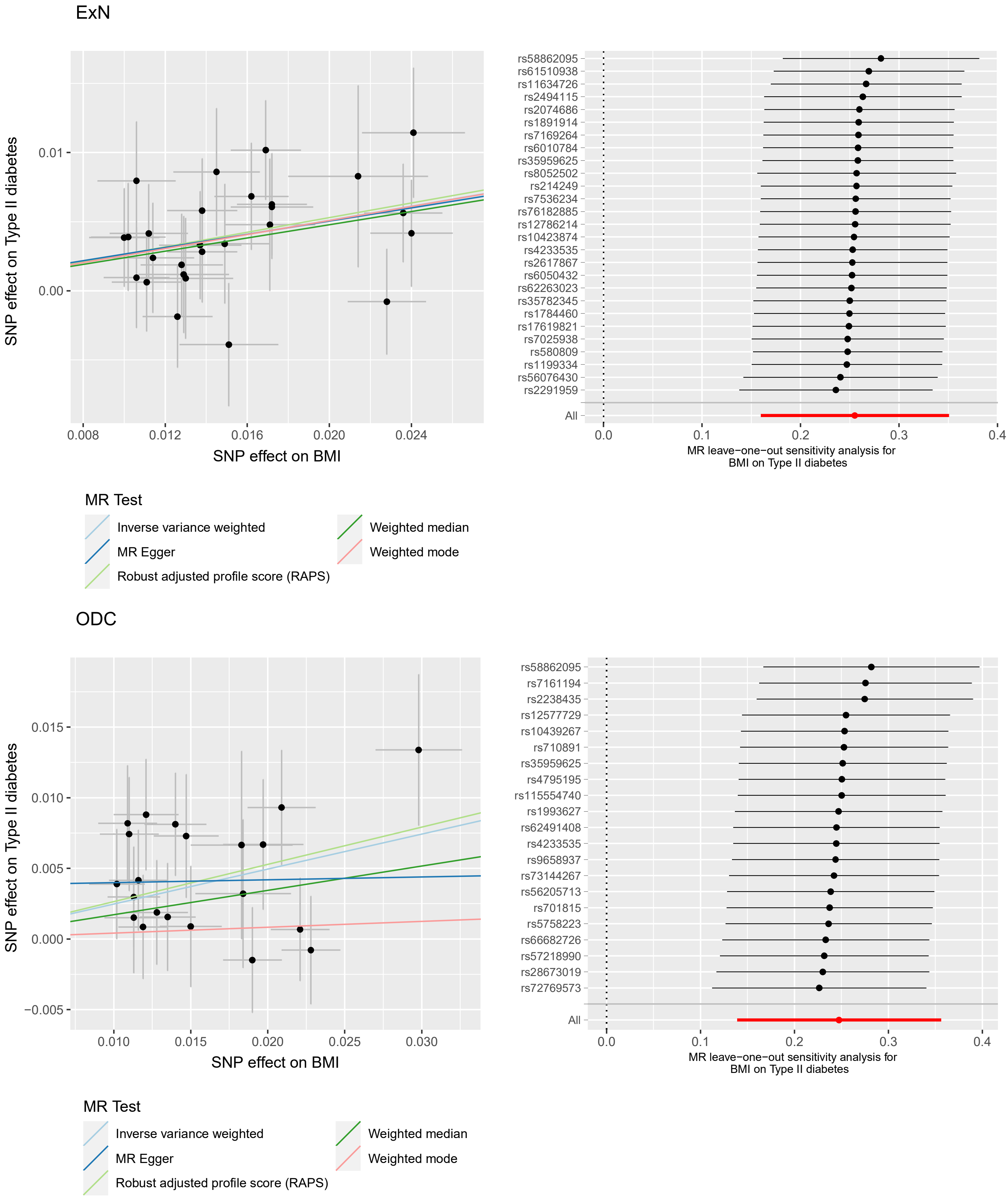

Supplementary Fig. 2. The scatter plots of cell-stratified genetic associations with BMI (x-axis) over genetic associations with different outcomes (y-axis) in the left part, and leave-one-out analysis plots in the right part. The displaying results for outcomes (a) sleep disorders, (b) ADHD, (c) gout, (d) osteoporosis, (e) coronary artery disease, (f) myocardial infarction and (g) type II diabetes.

(a) Sleep disorders

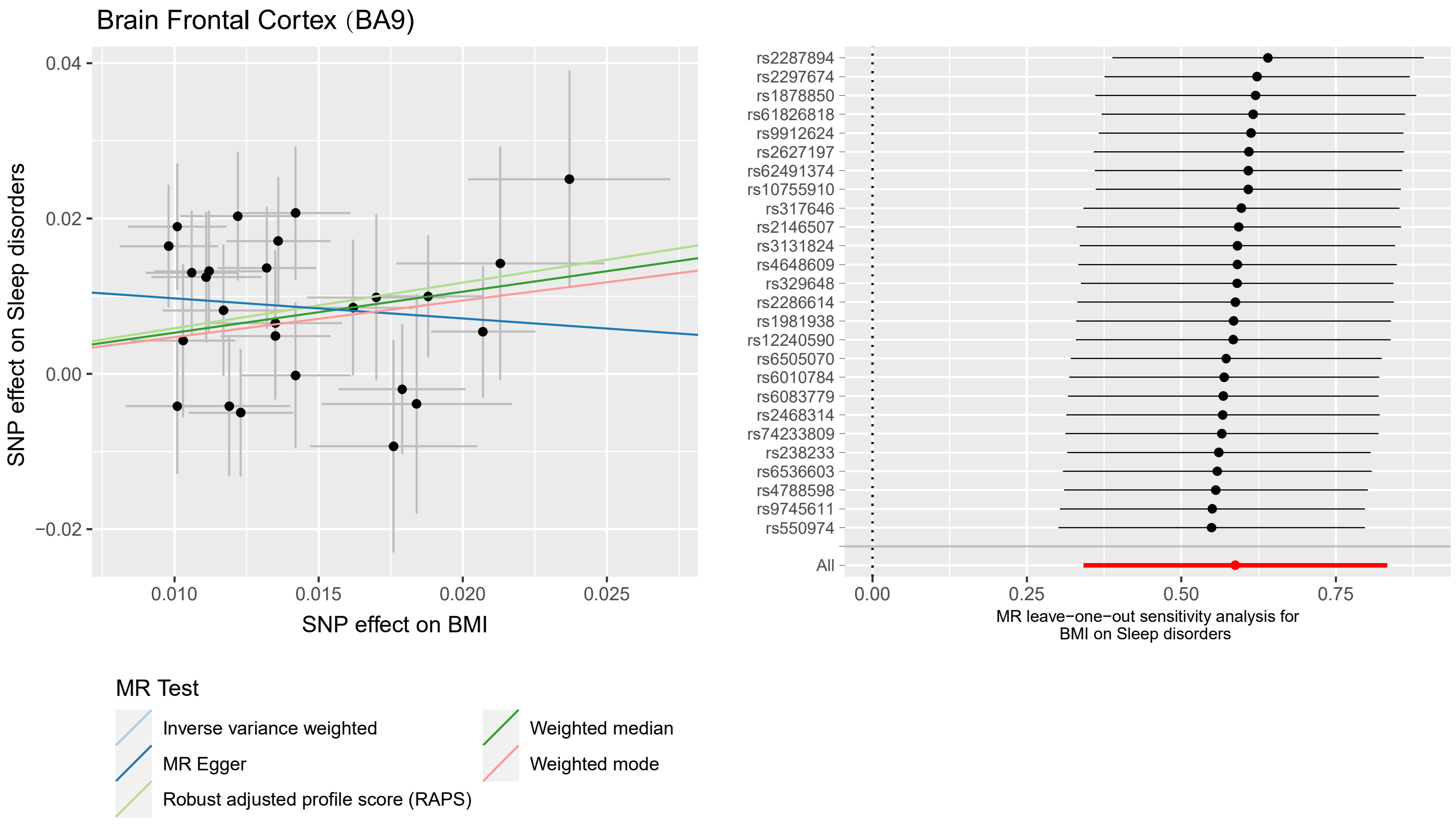

(b) Type II diabetes

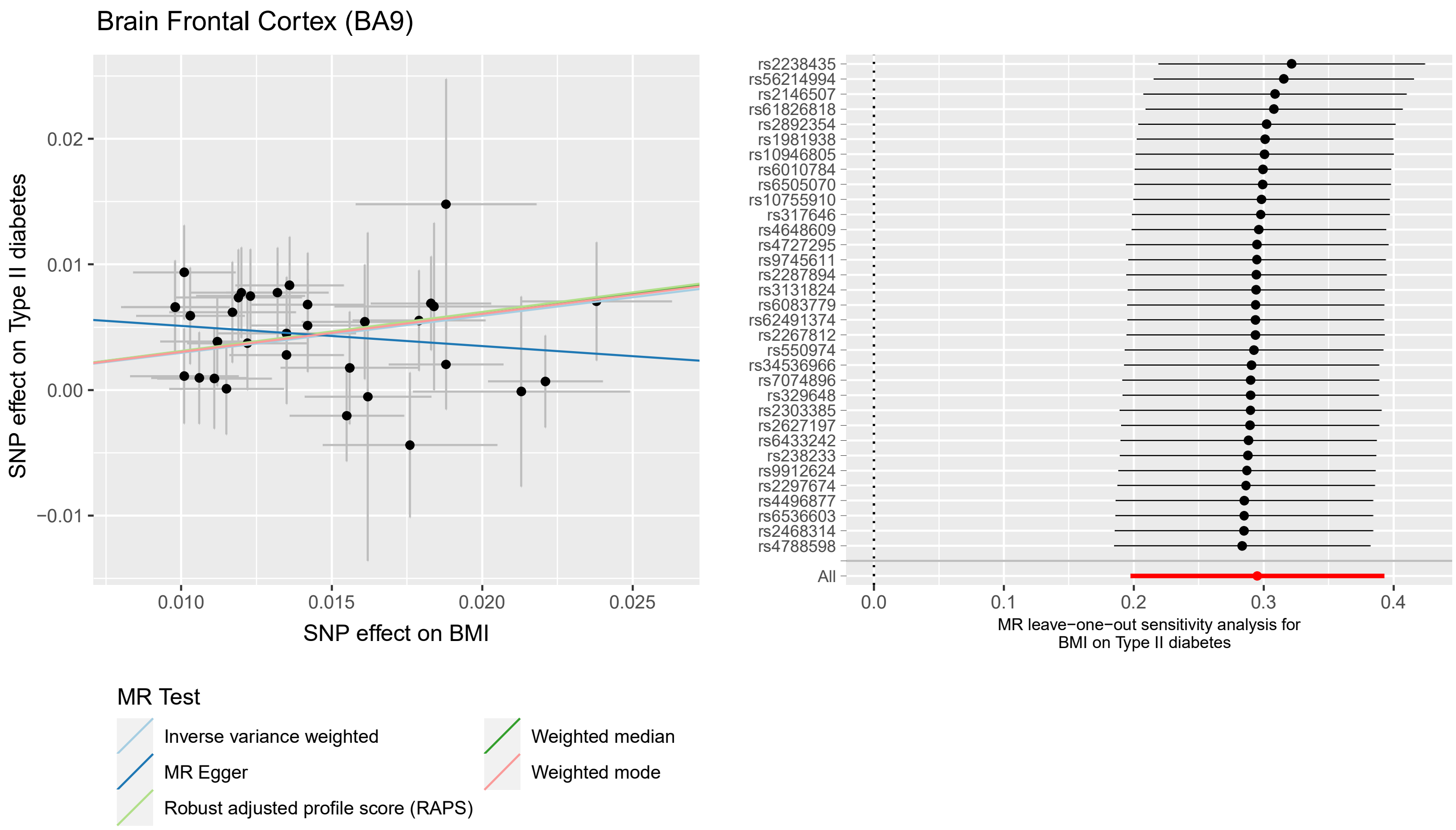

Supplementary Fig. 3. The scatter plots of tissue-stratified genetic associations with BMI (x-axis) over genetic associations with different outcomes (y-axis) in the left part, and leave-one-out analysis plots in the right part.The displaying results for outcomes (a) sleep disorders and (b) type II diabetes.

(a) Stroke

Astrocyte

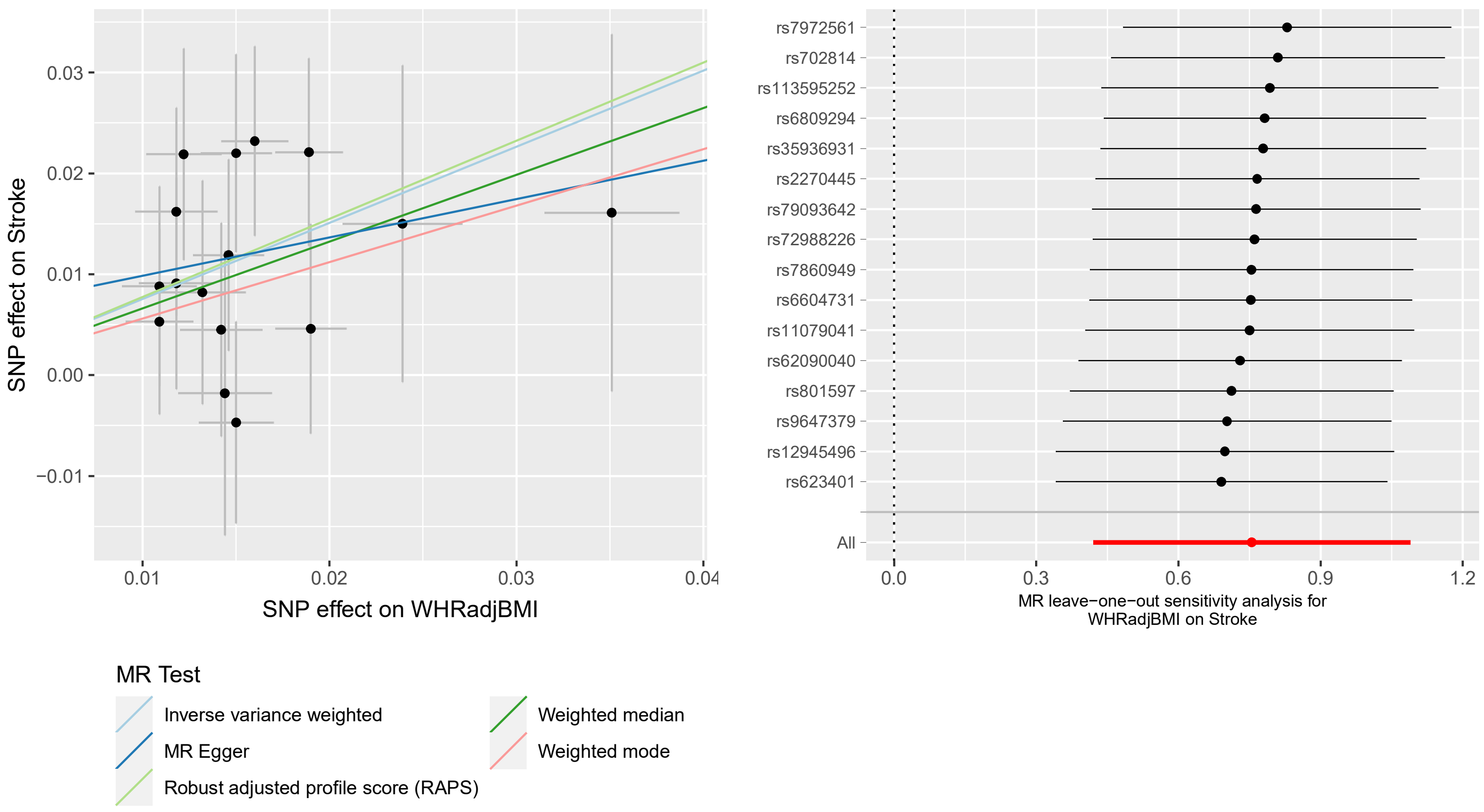

ExN

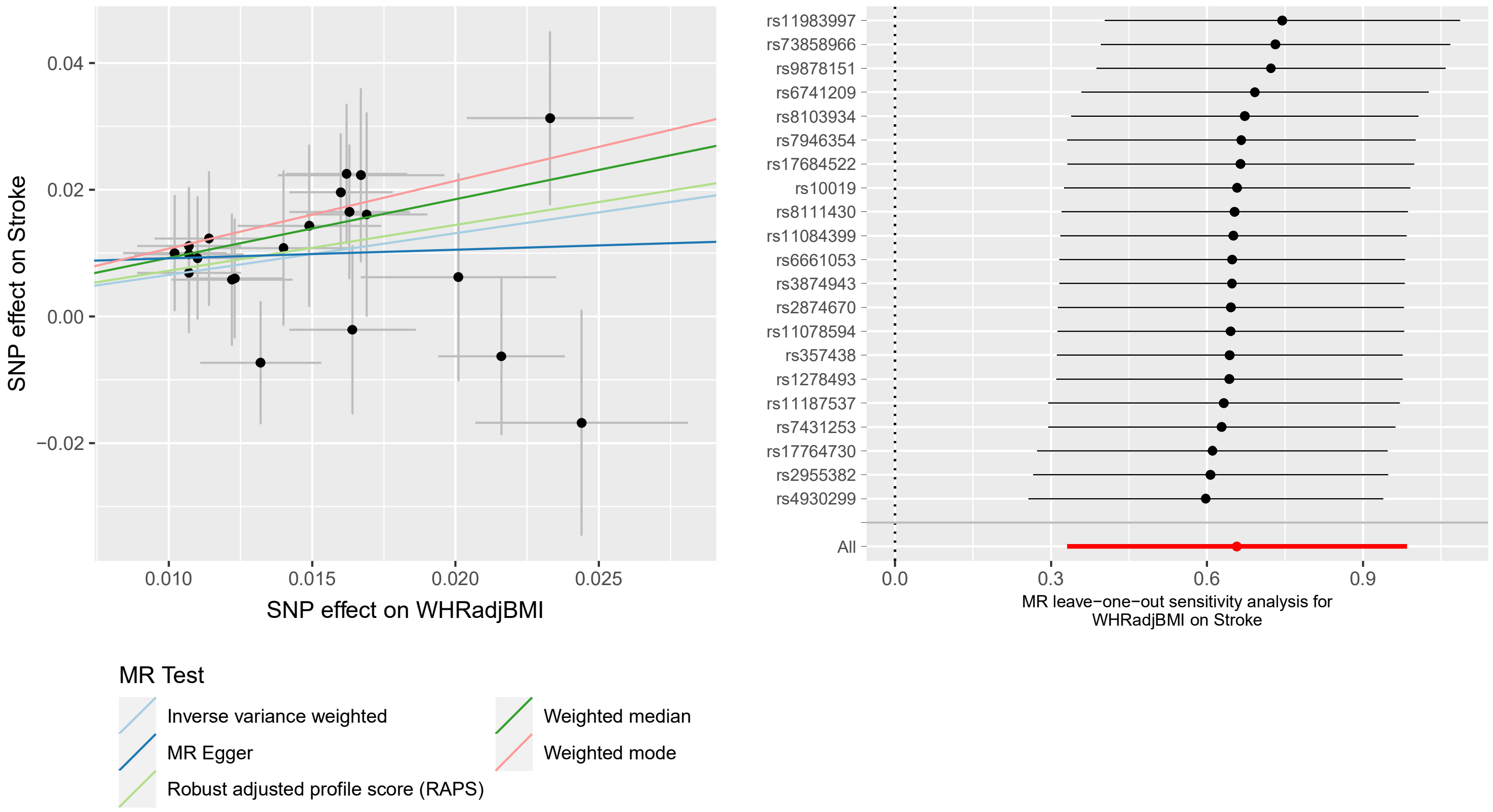

lnN

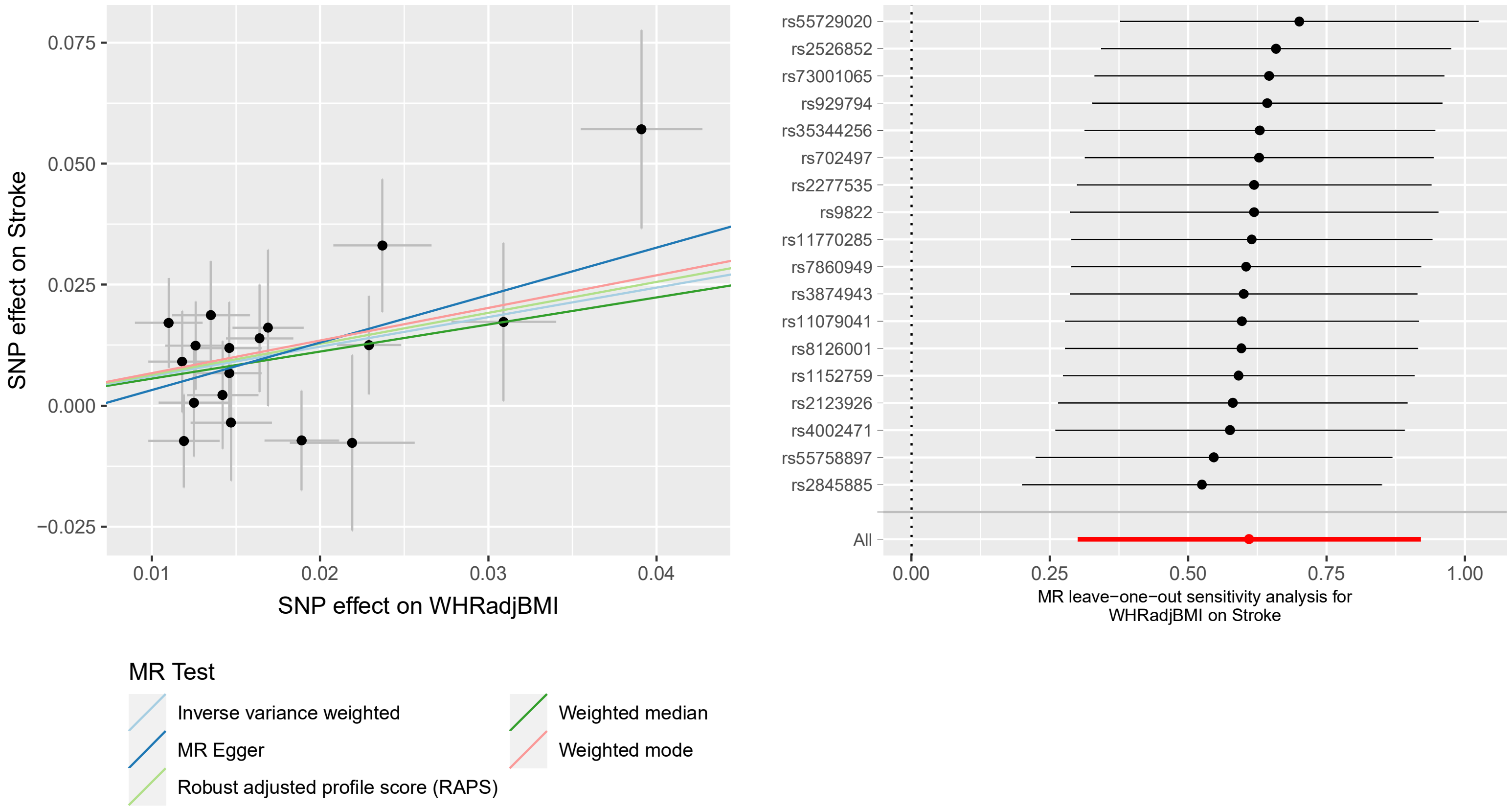
0.075
0.050
0.025
0.000
−0.025
SNP effect on Stroke
0.01
0.02
0.03
0.04
SNP effect on WHRadjBMI
MR Test
Inverse variance weighted
MR Egger
Robust adjusted profile score (RAPS)
Weighted median
Weighted mode
rs55729020
rs2526852
rs73001065
rs929794
rs35344256
rs702497
rs2277535
rs9822
rs11770285
rs7860949
rs3874943
rs11079041
rs8126001
rs1152759
rs2123926
rs4002471
rs55758897
rs2845885
All
0.00
0.25
0.50
0.75
1.00
MR leave−one−out sensitivity analysis for
WHRadjBMI on Stroke

(b) Coronary artery disease

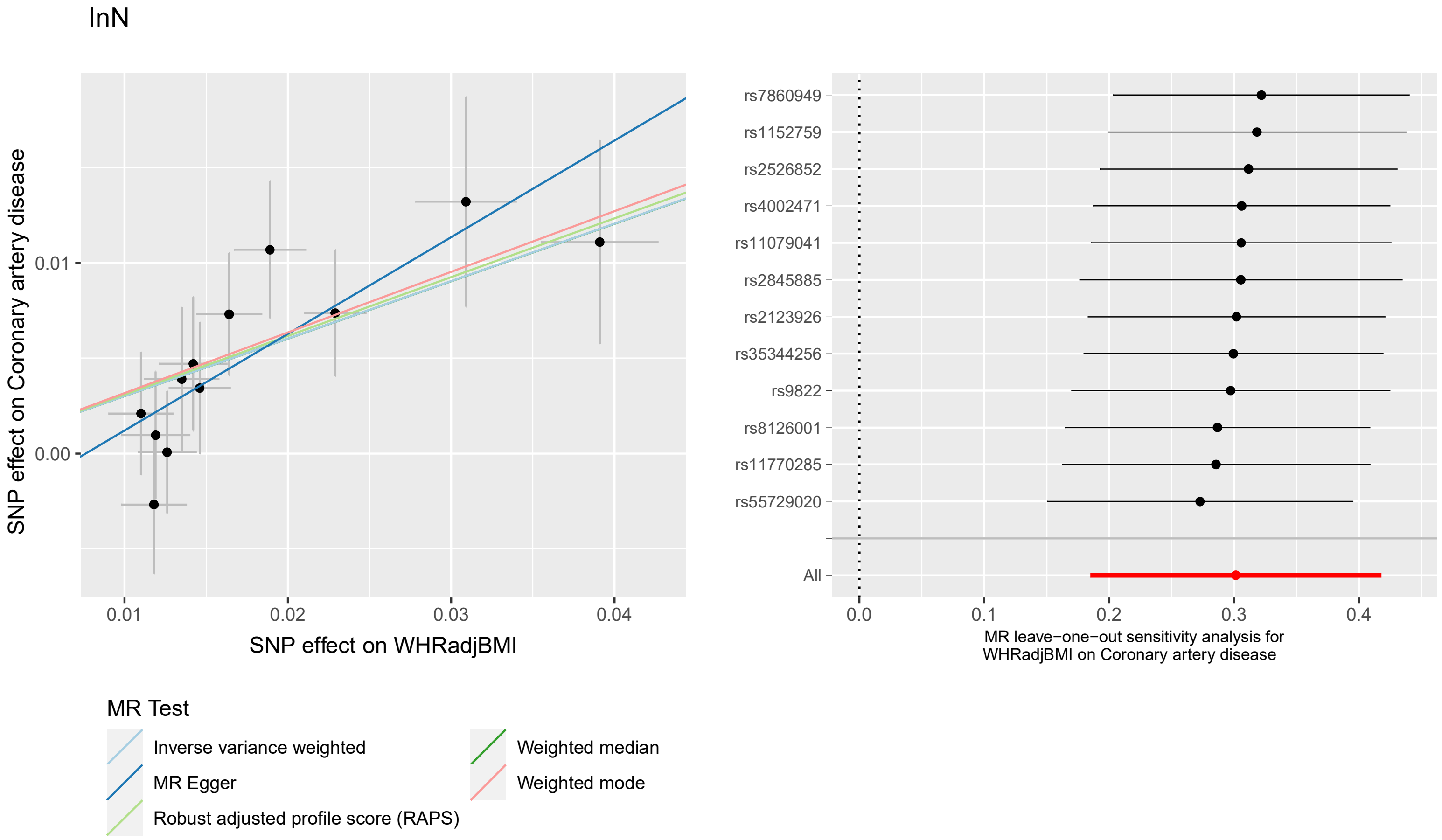

(c) Type II diabetes

InN

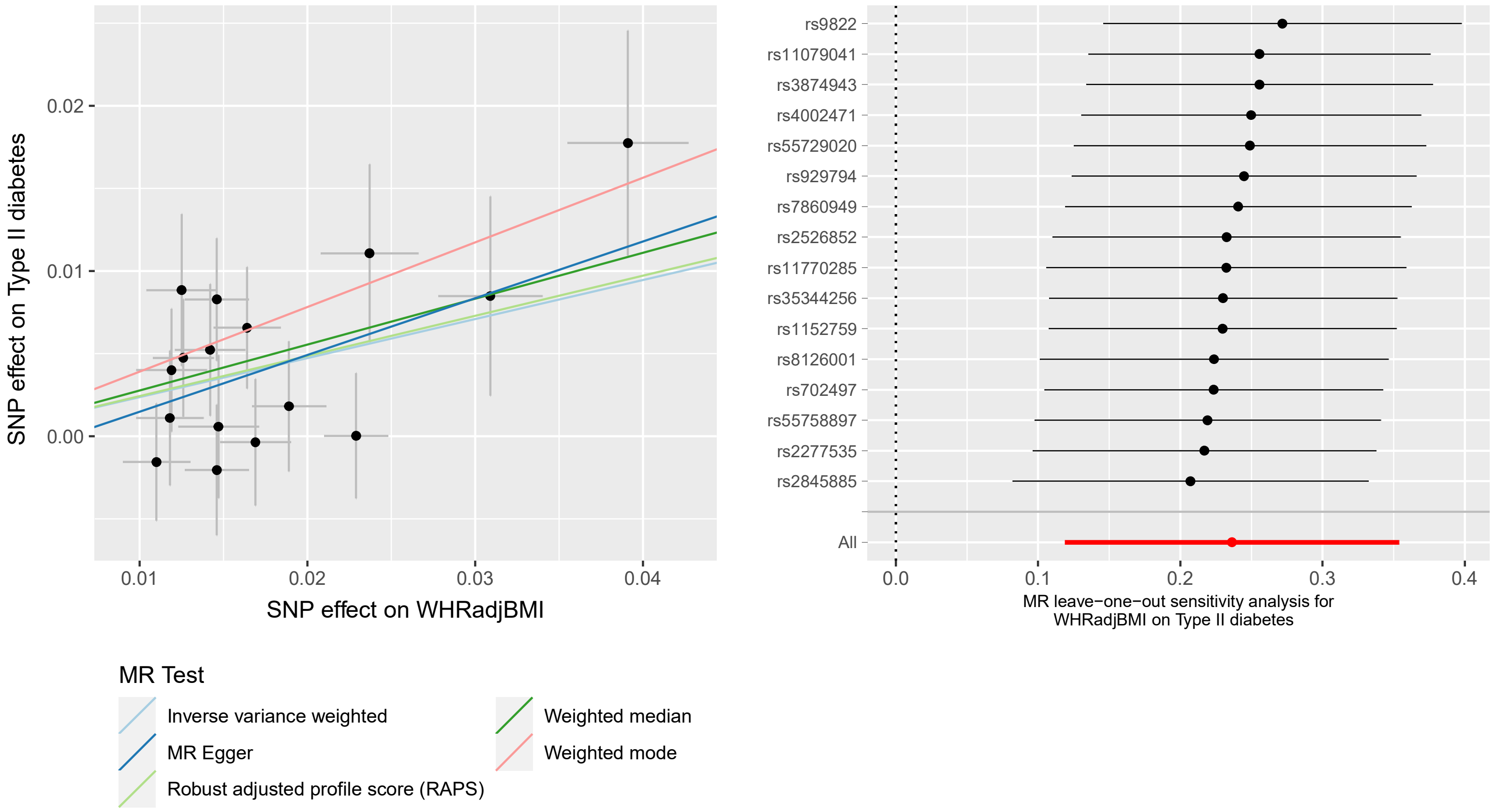

OPC

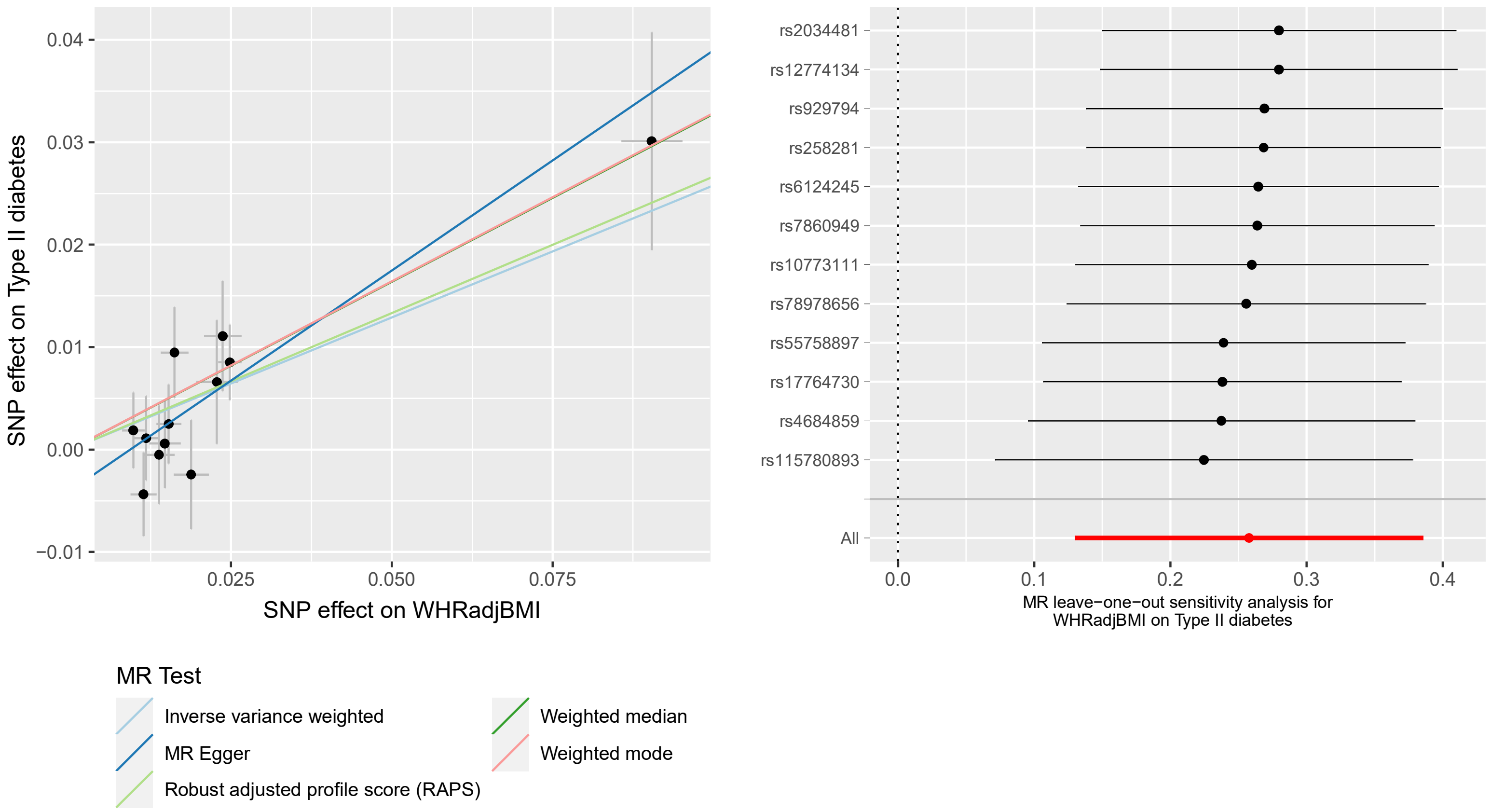

Supplementary Fig. 4. The scatter plots of cell-stratified genetic associations with WHRadjBMI (x-axis) over genetic associations with different outcomes (y-axis) in the left part, and leave-one-out analysis plots in the right part.The displaying results for outcomes (a) stroke, (b) coronary artery disease and (c) type II diabetes.

(a) Epilepsy

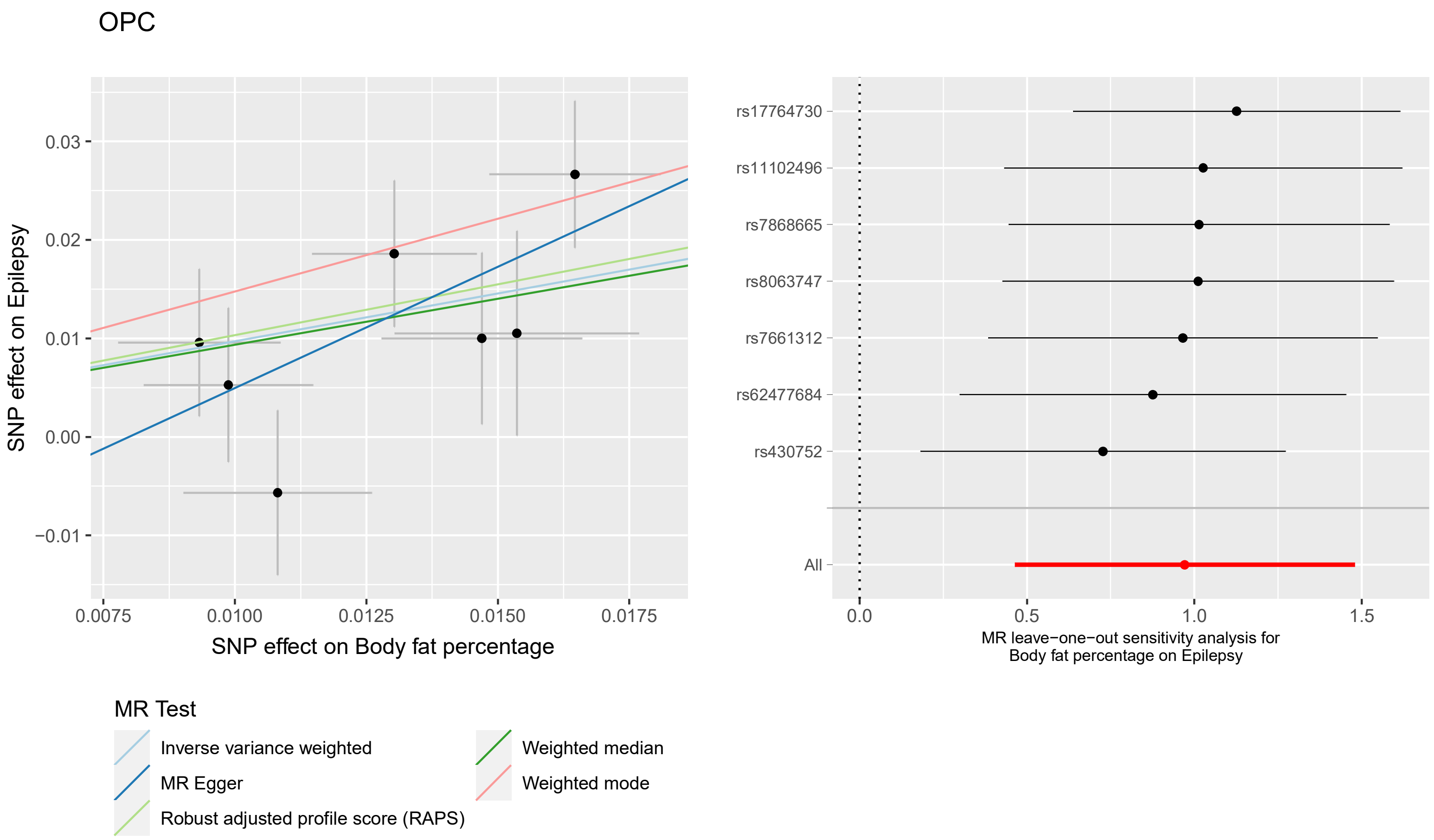
OPC
SNP effect on Epilepsy
0.03
0.02
0.01
0.00
−0.01
0.0075
0.0100
0.0125
0.0150
0.0175
SNP effect on Body fat percentage
rs17764730
rs11102496
rs7868665
rs8063747
rs7661312
rs62477684
rs430752
All
0.0
0.5
1.0
1.5
MR leave−one−out sensitivity analysis for
Body fat percentage on Epilepsy
MR Test
Inverse variance weighted
MR Egger
Robust adjusted profile score (RAPS)
Weighted median
Weighted mode

(b) ADHD

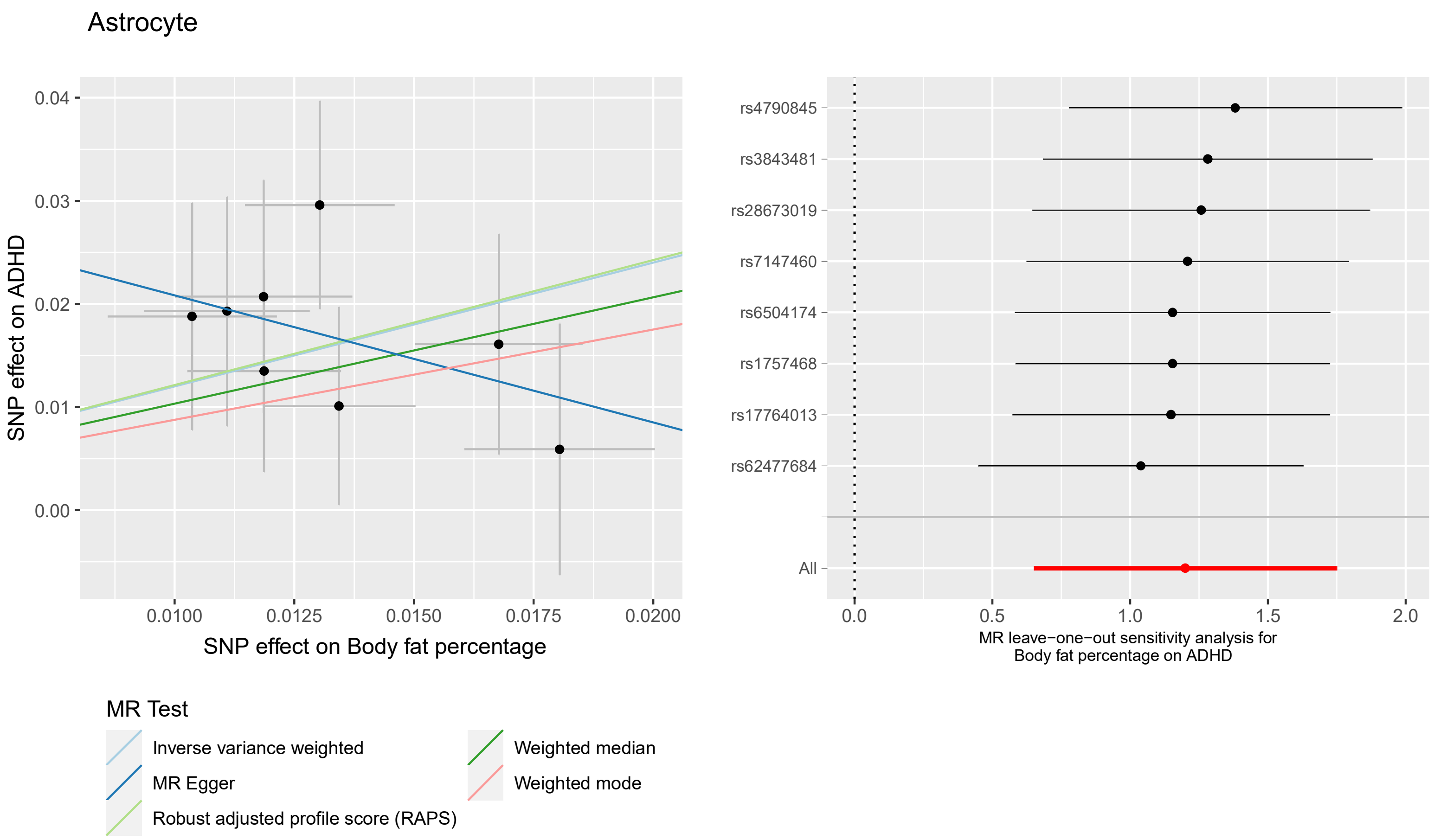
Astrocyte
SNP effect on ADHD
0.04
0.03
0.02
0.01
0.00
0.0100
0.0125
0.0150
0.0175
0.0200
SNP effect on Body fat percentage
rs4790845
rs3843481
rs28673019
rs7147460
rs6504174
rs1757468
rs17764013
rs62477684
All
0.0
0.5
1.0
1.5
2.0
MR leave−one−out sensitivity analysis for
Body fat percentage on ADHD
MR Test
Inverse variance weighted
MR Egger
Robust adjusted profile score (RAPS)
Weighted median
Weighted mode

(c) Schizophrenia

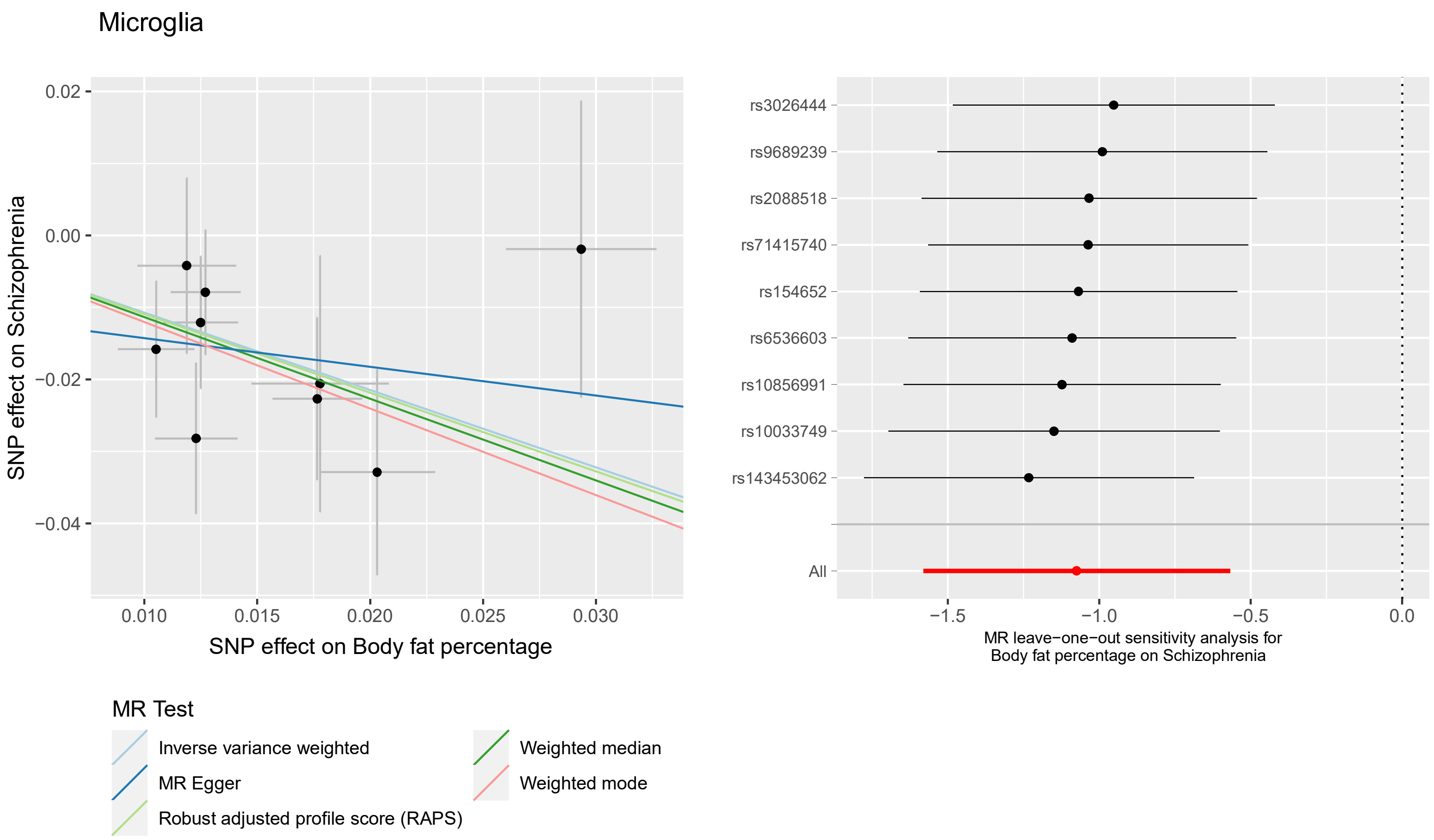

(d) Knee & hip osteoarthritis

ODC

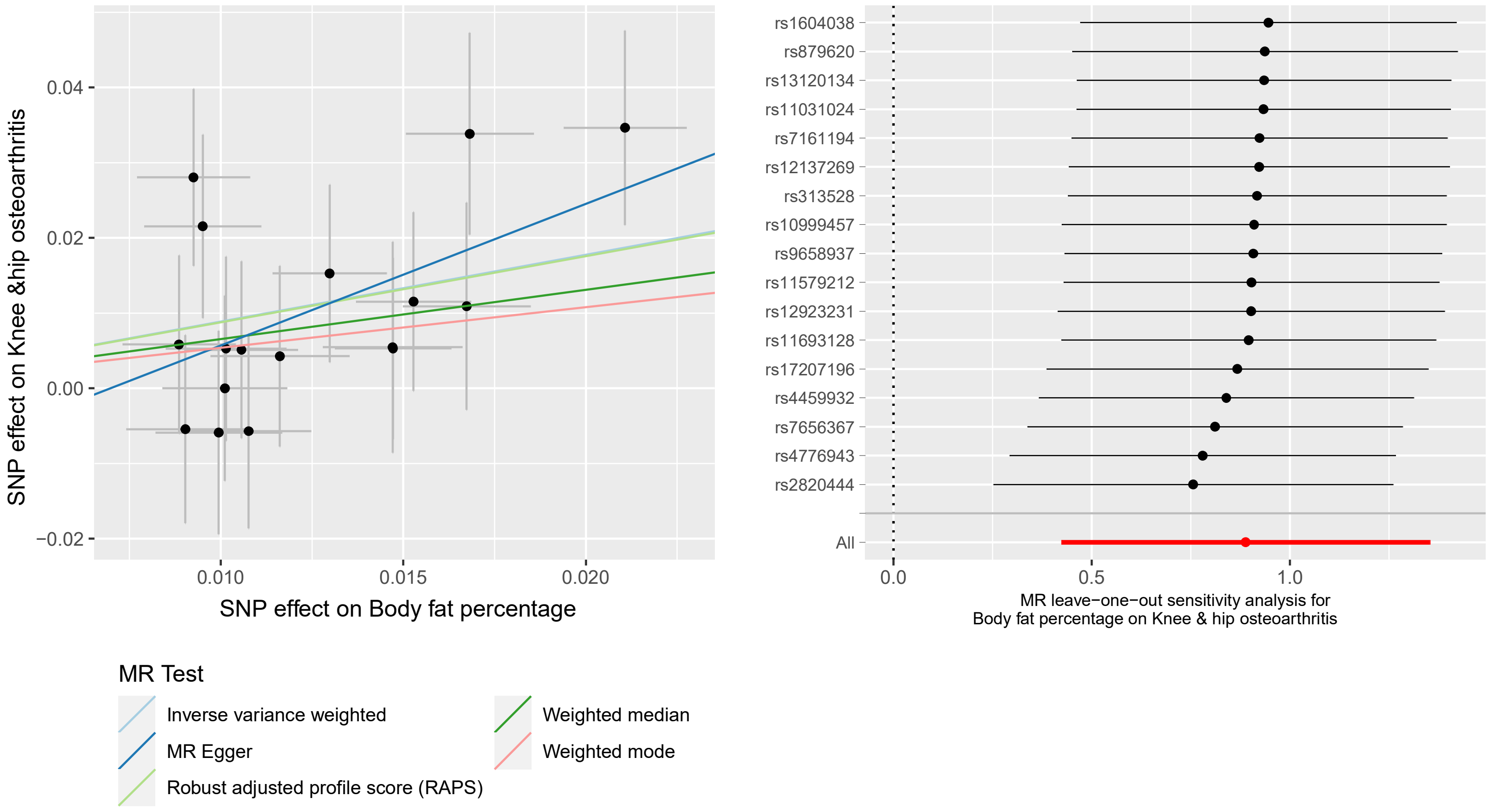

OPC

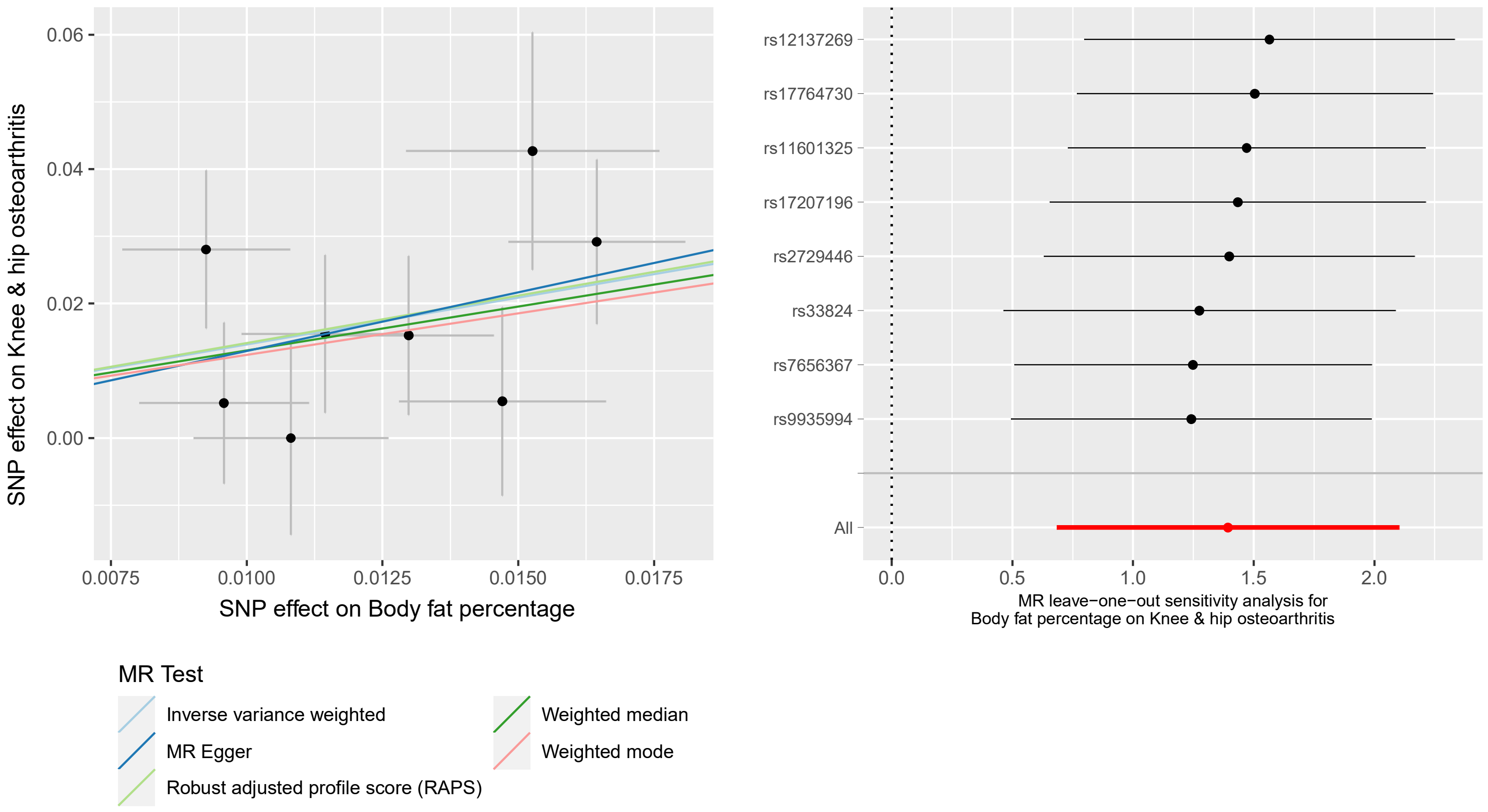

Supplementary Fig. 5. The scatter plots of cell-stratified genetic associations with body fat percentage (x-axis) over genetic associations with different outcomes (y-axis) in the left part, and leave-one-out analysis plots in the right part.The displaying results for outcomes (a) epilepsy, (b) ADHD, (c) schizophrenia and (d) knee & hip osteoarthritis.

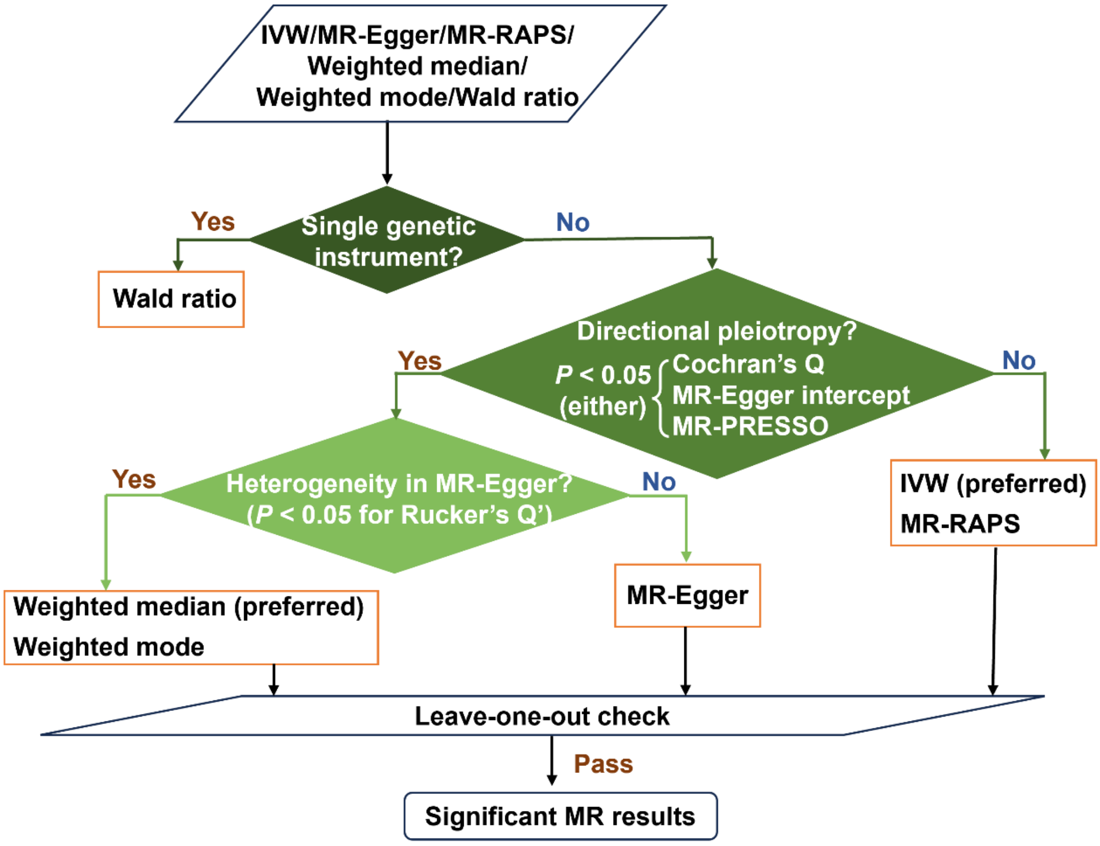

Supplementary Fig. 6. The flow chart showing how we selected main MR method by performing a series of pleiotropy and sensitivity analyses.
