# Supplementary material for: Revealing brain cell-stratified causality through dissecting causal variants according to their cell-type-specific effects on gene expression
Source: Nat Commun. 2024 Jun 7;15:4890. doi: 10.1038/s41467-024-49263-4 (PMC11161590; doi:10.1038/s41467-024-49263-4)
Supplement: Supplementary file 3 — Description of Additional Supplementary Files [file 41467_2024_49263_MOESM3_ESM.pdf]

**Description of Additional Supplementary Files**

**Supplementary Data 1.** A summary of data sources used in colocalization analyses.

**Supplementary Data 2.** Summary of variants and genes with strong evidence for colocalization (PPH4 > 0.8).

**Supplementary Data 3.** *P* values of two-sided paired Wilcoxon tests (corresponding to Fig. 2d and Supplementary Fig. 1). The gene expression comparison was conducted between reference cell types (row) and test cell types (column).

**Supplementary Data 4.** GWAS summary data of disease outcomes used in MR analysis.

**Supplementary Data 5.** MR results using all BMI associated variants.

**Supplementary Data 6.** Cell-stratified MR results for BMI.

**Supplementary Data 7.** Results of pleiotropy and sensitivity analyses for BMI based cell-stratified MR results.

**Supplementary Data 8.** Tissue-stratified MR results for BMI.

**Supplementary Data 9.** Results of pleiotropy and sensitivity analyses for BMI based tissue-stratified MR results.

**Supplementary Data 10.** Cell-stratified MR results for WHRadjBMI.

**Supplementary Data 11.** Results of pleiotropy and sensitivity analyses for WHRadjBMI based cell-stratified MR results.

**Supplementary Data 12.** Cell-stratified MR results for body fat percentage.

**Supplementary Data 13.** Results of pleiotropy and sensitivity analyses for body fat percentage based cell-stratified MR results.

**Supplementary Software 1.** The csMR software is an analytical tool for inferring cell-stratified causality with GWAS and single-cell eQTL data. Detailed installation and usage information can be found at https://github.com/rhhao/csMR.
