# Supplementary material for: Revealing brain cell-stratified causality through dissecting causal variants according to their cell-type-specific effects on gene expression
Source: Nat Commun. 2024 Jun 7;15:4890. doi: 10.1038/s41467-024-49263-4 (PMC11161590; doi:10.1038/s41467-024-49263-4)
Supplement: Supplementary file 5 — Supplementary Software 1 [file 41467_2024_49263_MOESM5_ESM.zip › csMR_v1.2/csMR_v1.2_readme.docx]

**csMR**

We introduce the Cell-Stratified Mendelian Randomization (csMR) framework to find cell-stratified causality of complex traits/diseases by integrating GWAS and single-cell eQTL data.

**Installation**

csMR can be downloaded by cloning this repository via the commands:

git clone <https://github.com/rhhao/csMR.git>

cd csMR

**Prerequisites**

1. Anaconda or Miniconda distribution

In order to void any issues with software versioning, csMR utilises conda environments to automatically install all necessary dependencies. If conda is not present on your system, please install anaconda or miniconda following the official instructions.

Once conda is ready, users can create an environment to install dependencies that csMR needs though the following commands:

conda install -c conda-forge mamba

mamba env create --file envs/envpy3.yml

conda activate csMR

2. R dependencies

csMR requires R packages phenoscanner and updated mr.raps (version 0.4.1). To install these libraries, run the following commands:

if (!require("devtools")) { install.packages("devtools") }

devtools::install_github("phenoscanner/phenoscanner", force = TRUE, lib="<your installation path to conda>/envs/csMR/lib/R/library")

devtools::install_github("qingyuanzhao/mr.raps", force = TRUE, lib="<your installation path to conda>/envs/csMR/lib/R/library")

(Notice: in order to successfully run csMR, we suggest to set your temporary R library paths by “export R_LIBS=<your installation path to conda>/envs/csMR/lib/R/library:$R_LIBS” before running csMR.)

3. PLINK

csMR requires PLINK (version 1.90) to calculate LD matrix. Follow the official instructions to install PLINK: https://www.cog-genomics.org/plink2/.

**Getting Started**

1. Data preparation

csMR requires summary-level GWAS and QTL data that consist of at least 8 required columns for GWAS and 9 required columns for QTL. Other columns will be ignored during processing. ***Notice: the names of required columns MUST be consistent with the following headers**, while the order can be inconsistent. The required data format is presented as follows:

GWAS summary stats:

| SNP | A1 | A2 | MAF | BETA | SE | P | N |
| --- | --- | --- | --- | --- | --- | --- | --- |
| rs1 | A | T | 0.125 | 0.166 | 0.178 | 0.734 | 480000 |
| … | … | … | … | … | … | … | … |
| rs256 | C | G | 0.201 | 0.037 | 0.079 | 0.349 | 480000 |

QTL summary stats:

| SNP | GENE | A1 | A2 | MAF | BETA | SE | P | N |
| --- | --- | --- | --- | --- | --- | --- | --- | --- |
| rs1 | geneY | A | T | 0.359 | -0.039 | 0.094 | 0.082 | 215 |
| … | … | … | … | … | … | … | … | … |
| rs256 | geneX | C | G | 0.229 | 0.064 | 0.012 | 0.779 | 215 |

2. Modify the config.yml file

Before running csMR, a configuration file needs to be modified to specify the input data and advanced parameters. An example is provided in the config.yml file that will process the example data obtained by further [download](http://www.bigc.online/csMR/index.php). The demo data are ready to use after decompressing into the installation directory of csMR.

Reference genome data are also required to run example data. Users can download reference_genome_1000G_EUR [here](http://www.bigc.online/csMR/index.php). The genome data are ready to use after decompressing into the csMR/data directory.

3. Run csMR

Now you have everything csMR needs, then you can run the analysis workflow under your installation directory by:

snakemake -s work_flow.snakefile --configfile config.yml -j

We recommend running with -j as it will use all available cores. Specifying -j 4 will use up to 4 cores.

4. Inspect the output

The results of colocalization and MR will be placed in <your output dir>/COLCO and <your output dir>/MR respectively.

**Contributors**

Ruo-Han Hao (Xi’an Jiaotong University, China)

Feng Jiang (Xi’an Jiaotong University, China)

Tian-Pei Zhang (Xi’an Jiaotong University, China)

Jun-Hui Liu (Xi’an Jiaotong University, China)

**Contact**

Please create an issue on the github repo if you encounter any problems. You can also contact the developers through email: ruohanhao(at)xjtu.edu.cn.
