# Supplementary material for: Revealing brain cell-stratified causality through dissecting causal variants according to their cell-type-specific effects on gene expression
Source: Nat Commun. 2024 Jun 7;15:4890. doi: 10.1038/s41467-024-49263-4 (PMC11161590; doi:10.1038/s41467-024-49263-4)
Supplement: Supplementary file 6 — Reporting Summary [file 41467_2024_49263_MOESM6_ESM.pdf]

nature portfolio

Corresponding author(s): Tie-Lin Yang, Yan Guo

Last updated by author(s): Apr 26, 2024

Reporting Summary

Nature Portfolio wishes to improve the reproducibility of the work that we publish. This form provides structure for consistency and transparency in reporting. For further information on Nature Portfolio policies, see our Editorial Policies and the Editorial Policy Checklist.

Statistics

For all statistical analyses, confirm that the following items are present in the figure legend, table legend, main text, or Methods section.

n/a	Confirmed	
☐	☒	The exact sample size (*n*) for each experimental group/condition, given as a discrete number and unit of measurement
☐	☒	A statement on whether measurements were taken from distinct samples or whether the same sample was measured repeatedly
☐	☒	The statistical test(s) used AND whether they are one- or two-sided<br>*Only common tests should be described solely by name; describe more complex techniques in the Methods section.*
☒	☐	A description of all covariates tested
☐	☒	A description of any assumptions or corrections, such as tests of normality and adjustment for multiple comparisons
☐	☒	A full description of the statistical parameters including central tendency (e.g. means) or other basic estimates (e.g. regression coefficient) AND variation (e.g. standard deviation) or associated estimates of uncertainty (e.g. confidence intervals)
☐	☒	For null hypothesis testing, the test statistic (e.g. *F*, *t*, *r*) with confidence intervals, effect sizes, degrees of freedom and *P* value noted<br>*Give P values as exact values whenever suitable.*
☒	☐	For Bayesian analysis, information on the choice of priors and Markov chain Monte Carlo settings
☒	☐	For hierarchical and complex designs, identification of the appropriate level for tests and full reporting of outcomes
☐	☒	Estimates of effect sizes (e.g. Cohen's *d*, Pearson's *r*), indicating how they were calculated

Our web collection on statistics for biologists contains articles on many of the points above.

Software and code

Policy information about availability of computer code

Data collection	No software was used to collect data.
Data analysis	The custom code is available in GitHub repository: https://github.com/rhhao/csMR. Publicly available software and packages used in this work include: snakemake 7.18.2, PLINK 1.9, R 4.2.2 and R packages coloc (v5.1.0), TwoSampleMR (v0.5.6), MRPRESSO (v1.0), RadialMR (v1.0), phenoscanner (v1.0).

For manuscripts utilizing custom algorithms or software that are central to the research but not yet described in published literature, software must be made available to editors and reviewers. We strongly encourage code deposition in a community repository (e.g. GitHub). See the Nature Portfolio guidelines for submitting code & software for further information.

Data

Policy information about availability of data

All manuscripts must include a data availability statement. This statement should provide the following information, where applicable:

- Accession codes, unique identifiers, or web links for publicly available datasets
- A description of any restrictions on data availability
- For clinical datasets or third party data, please ensure that the statement adheres to our policy

The GWAS summary data for BMI and WHRadjBMI can be accessed from the GIANT data portal (https://portals.broadinstitute.org/collaboration/giant/index.php/GIANT_consortium_data_files). The body fat percentage GWAS data can be obtained from the MRC IEU OpenGWAS database (https://gwas.mrcieu.ac.uk/datasets/

ukb-b-8909/). Brain single-cell eQTL data were obtained from https://doi.org/10.5281/zenodo.5543734. GTEx v8 data were downloaded from https://www.gtexportal.org/home/datasets. All GWAS summary statistics for disease outcomes used in this study are publicly available for download from the resources summarized in Supplementary Table 3.

Research involving human participants, their data, or biological material

Policy information about studies with human participants or human data. See also policy information about sex, gender (identity/presentation), and sexual orientation and race, ethnicity and racism.

Reporting on sex and gender	All our analyses were based on publicly available data, and all available participants were included. Detailed sample information can be obtained from the original studies (Supplementary Tables 1 and 3). Briefly, according to their description of study samples, sex differences were not significantly observed.
Reporting on race, ethnicity, or other socially relevant groupings	All our analyses were based on publicly available data. To avoid ethnic bias in colocalization and Mendelian randomization analyses, we obtained GWAS data that involve participants of European ancestry or > 80% of the samples are European.
Population characteristics	All our analyses were based on publicly available data, and all available participants were included. Detailed sample information can be obtained from the original studies. GWAS data of obesity-related traits were derived from the Genetic Investigation of Anthropometric Traits (GIANT) consortium and the UK Biobank (UKB) involving subjects with anthropometric traits. GWAS data of 18 complex diseases involve participants of cases and controls, where the cases were diagnosed with disease conditions at the time of recruitment.
Recruitment	All our analyses were based on publicly available data, and all available participants were included. The recruitment criteria vary from dataset to dataset. Detailed sample inclusion information can be obtained from the original studies.
Ethics oversight	The GWAS and eQTL summary data we used are all publicly released, and ethical approval was provided in each study.

Note that full information on the approval of the study protocol must also be provided in the manuscript.

Field-specific reporting

Please select the one below that is the best fit for your research. If you are not sure, read the appropriate sections before making your selection.

☒ Life sciences ☐ Behavioural & social sciences ☐ Ecological, evolutionary & environmental sciences

For a reference copy of the document with all sections, see nature.com/documents/nr-reporting-summary-flat.pdf

Life sciences study design

All studies must disclose on these points even when the disclosure is negative.

Sample size	All our analyses were based on publicly available data. We used the largest available GWAS summary data and brain eQTL data in this work, and all available samples were included. The sample sizes of eQTL data are 192 for single-cell dataset, and ~205 for bulk datasets. The sample sizes of GWAS data ranged from 9,954 to 694,649. The detailed information are summarized in Supplementary Tables 1 and 3.
Data exclusions	For selecting genetic instruments, we removed palindromic SNPs with intermediate allele frequencies (>0.42) and outlier pleiotropic SNPs via heterogeneity test (modified Q-statistics) using RadialMR with the P value threshold of 0.05 to satisfy the strong instrument criteria for Mendelian randomization analyses.
Replication	We used the largest GWAS summary data in this work that are also publicly available to researchers, therefore, no replication is included.
Randomization	This is not relevant to our study as it is not a randomized controlled trial. However, we implemented Mendelian randomization analyses that are based on the assumption that genetic variants are randomly allocated at conception and are relatively independent of self-selected behaviors and established well before the onset of disease.
Blinding	In this study, we only used summary-level genetic association data derived from different studies, and individual-level information is not required. So blinding is not relevant.

Reporting for specific materials, systems and methods

We require information from authors about some types of materials, experimental systems and methods used in many studies. Here, indicate whether each material, system or method listed is relevant to your study. If you are not sure if a list item applies to your research, read the appropriate section before selecting a response.

Materials & experimental systems

n/a	Involved in the study
☒	☐ Antibodies
☒	☐ Eukaryotic cell lines
☒	☐ Palaeontology and archaeology
☒	☐ Animals and other organisms
☒	☐ Clinical data
☒	☐ Dual use research of concern
☒	☐ Plants

Methods

n/a	Involved in the study
☒	☐ ChIP-seq
☒	☐ Flow cytometry
☒	☐ MRI-based neuroimaging
